# The complete chloroplast genomes of *Tetrastigma hemsleyanum* (Vitaceae) from different regions of China: molecular structure, comparative analysis and development of DNA barcodes for its geographical origin discrimination

**DOI:** 10.1186/s12864-022-08755-7

**Published:** 2022-08-26

**Authors:** Shujie Dong, Manjia Zhou, Jinxing Zhu, Qirui Wang, Yuqing Ge, Rubin Cheng

**Affiliations:** 1 The First Affiliated Hospital of Zhejiang Chinese Medical University, Hangzhou, China; 2 School of Pharmaceutical Sciences, Zhejiang Chinese Medical University, Hangzhou, China; 3 Bureau of Agricultural and Rural Affairs of Suichang, Suichang, China; 4 Academy of Chinese Medical Science, Zhejiang Chinese Medical University, Hangzhou, China

**Keywords:** *Tetrastigma hemsleyanum*, Chloroplast genome, Phylogenetic relationships, Nucleotide diversity, DNA barcoing markers, Geographical origins

## Abstract

**Background:**

*Tetrastigma hemsleyanum* is a valuable traditional Chinese medicinal plant widely distributed in the subtropical areas of China. It belongs to the Cayratieae tribe, family Vitaceae, and exhibited significant anti-tumor and anti-inflammatory activities. However, obvious differences were observed on the quality of *T. hemsleyanum* root from different regions, requiring the discrimination strategy for the geographical origins.

**Result:**

This study characterized five complete chloroplast (cp) genomes of *T. hemsleynum* samples from different regions, and conducted a comparative analysis with other representing species from family Vitaceae to reveal the structural variations, informative markers and phylogenetic relationships. The sequenced cp genomes of *T. hemsleyanum* exhibited a conserved quadripartite structure with full length ranging from 160,124 bp of Jiangxi Province to 160,618 bp of Zhejiang Province. We identified 112 unique genes (80 protein-coding, 28 tRNA and 4 rRNA genes) in the cp genomes of *T. hemsleyanum* with highly similar gene order, content and structure. The IR contraction/expansion events occurred on the junctions of *ycf1*, *rps19* and *rpl2* genes with different degrees, causing the differences of genome sizes in *T. hemsleyanum* and Vitaceae plants. The number of SSR markers discovered in *T. hemsleyanum* was 56–57, exhibiting multiple differences among the five geographic groups. Phylogenetic analysis based on conserved cp genome proteins strongly grouped the five *T. hemsleyanum* species into one clade, showing a sister relationship with *T. planicaule*. Comparative analysis of the cp genomes from *T. hemsleyanum* and Vitaceae revealed five highly variable spacers, including 4 intergenic regions and one protein-coding gene (*ycf1*). Furthermore, five mutational hotspots were observed among *T. hemsleyanum* cp genomes from different regions, providing data for designing DNA barcodes *trnL* and *trnN*. The combination of molecular markers of *trnL* and *trnN* clustered the *T. hemsleyanum* samples from different regions into four groups, thus successfully separating specimens of Sichuan and Zhejiang from other areas.

**Conclusion:**

Our study obtained the chloroplast genomes of *T. hemsleyanum* from different regions, and provided a potential molecular tracing tool for determining the geographical origins of *T. hemsleyanum*, as well as important insights into the molecular identification approach and and phylogeny in *Tetrastigma* genus and Vitaceae family.

**Supplementary Information:**

The online version contains supplementary material available at 10.1186/s12864-022-08755-7.

## Introduction

*Tetrastigma hemsleyanum* Diels et Gilg (*T. hemsleyanum*) is a unique and valuable Chinese medicinal herb belonging to the tribe cayratieae of family Vitaceae. It is mainly distributed around the central, eastern, southern and southwestern provinces of China [1]. As a customary Chinese medicine, *T. hemsleyanum* has been recorded in the Zhejiang Provincial Standards of Processing Chinese Crud Drugs (2015) with the therapeutic effects of heat-clearing, toxicity-removing, promoting blood circulation and pain relief. The roots of *T. hemsleyanum* have been used traditionally to treat high fever, pneumonia, hepatitis, and multiple types of cancers alone or in combination with other herbal medicines [2]. Recent pharmacological investigations have demonstrated that *T. hemsleyanum* possesses anti-inflammatory, anti-virus, anti-tumor and immunomodulatory effects, which could be attributed to the active components of flavonoids, polysaccharides, terpenoids and alkaloids [3]. Total flavones of *T. hemsleyanum* could inhibit the proliferation and induce apoptosis of breast cancer cells MDA-MB-468 and MCF-7 by inhibiting the expression of p-p42/44 and blocking MAPK signaling pathway [4]. In addition, dietary flavone from *T. hemsleyanum* vine was also found to trigger human lung adenocarcinoma apoptosis via autophagy [5]. The extracts from *T. hemsleyanum* leaves exhibited protective effects against acrylamide induced toxicity both in HepG2 cells and *Caenorhabditis elegans* via regulating DAF-16/FOXO signaling pathway [6]. Furthermore, the polysaccharide isolated from *T. hemsleyanum* enhanced the immune responses in both OVA-immunized C57BL/6 mice and Lewis lung carcinoma bearing mice through activating TLR4 signaling pathway [7]. The phenolic contents of *T. hemsleyanum* leaves exhibited significant antioxidant activities, indicating the possibility of developing *T. hemsleyanum* leaves as functional foods [8]. Moreover, the herbal formula Hua Shi Xuan Fei mixture mainly composed of *T. hemsleyanum* has been approved by Zhejiang Provincial Drug Administration for clinical treatment of COVID-19, which played important roles in fighting the epidemic [9]. The high medicinal and economic value of *T. hemsleyanum* makes its further genetic and phylogenetic investigation necessary.

The roots of *T. hemsleyanum* were one of the most popular Chinese folk medicines in herbal market with the common name Sanyeqing. The genus *Tetrastigma* contained more than 100 species of climbers basically distributed in the tropics and subtropics of Asia, and a few species extending to Australia [10]. The *Tetrastigma* climbers have also attracted a ton of attention due to its unique host-parasite association with Rafflesiaceae, which possess the largest flowers in the world [11]. The great number of *Tetrastigma* species provided rich resources of root tubers for the potential counterfeits for *T. hemsleyanum*. Due to the lack of typical identification characteristics, it is difficult to distinguish roots of *T. hemsleyanum* from those adulterants of climbers in the genus of *Tetrastigma* and other closely related species. The increased number of counterfeit products and substitutes of *T. hemsleyanum* in the herbal medicine market have seriously harmed the clinical safety and effectiveness of *T. hemsleyanum* and significantly threatened its healthy development [12]. For instance, the root tubers of toxic *Aconitum carmichaeli* have been often sold as adulterants of *T. hemsleyanum* in medicine markets owing to high profit in Zhejiang Province, which caused many serious poisoning incidents. Furthermore, significant differences have been indicated in chemical compositions and therapeutic values of medicinal plants from different regions, which could be attributed to genetic varieties and growth environment [13]. *T. hemsleyanum* was widely distributed in the tropical to subtropical areas of China with multiple varieties and large gaps in the yield and quality of root tubers. The molecular investigations of *T. hemsleyanum* based on ISSR and SRAP analysis revealed the high genetic diversity at the level of species and low diversity in populations [14]. The *T. hemsleyanum* samples from Zhejiang and Fujian Province exhibited the highest contents of total flavonoids and strongest inhibitory activities against HepG2 cells, further confirming the crucial role of the geographical origins [15]. Numerous different expressed transcripts and multiple differentially accumulated metabolites mainly involved in phenylpropane and flavonoid biosynthesis have been found between two ecotypes of *T. hemsleyanum* from different regions [16]. In addition, the starch characterizations of *T. hemsleyanum* tuber roots from different origins showed significant differences on the granule diameter, amylose content and peak gelatinization temperature, from Zhejiang and Guangxi province [17]. Although the unique growth environment of different regions has great and profound influence on species, the diversity of *T. hemsleyanum* germplasm resources is beyond doubt, and their interaction determines the quality of *T. hemsleyanum*. The relationship of geographical difference and genetic difference of germplasm is of great significance. Therefore, authenticity and traceability of geographical origin of *T. hemsleyanum* were imperative and imminent for its quality and medicinal values to prevent mislabeling. The rapid and accurate discrimination of geographical origin of root from *T. hemsleyanum* would be conducive to the breeding and brand establishment of superior varieties. The growing demand of identifying the provenances of decoction pieces of *T. hemsleyanum* required effective approaches for geographical origin determination, which would significantly contribute to the further development and clinical applications of *T. hemsleyanum*.

The chloroplast (cp), which performs photosynthesis as well as harbors lots of other metabolic pathways, is an important organelle in plants and is generally non-recombinant and uniparentally inherited [18, 19]. In most angiosperms, chloroplast genome is generally double stranded and circular and the size varies from 120 kb to 160 kb [20]. The structure of chloroplast genome is highly conservated, consisting of a small single copy region (SSC), a large single copy region (LSC) and a pair of inverted repeat regions (IR) [21]. The cp genome harbors many different gene loci and non-coding regions containing relatively large amount of DNA sequence information, which has been widely considered as a powerful tool for phylogenetic analysis and further development of species identification and restoration strategies [22]. A great number of cp genomes have been determined and applied in phylogenetic reconstructions from population genetics to investigate the sequence evolution and perform deep divergence analysis at a genera and family level. Comparative chloroplast genome analysis of six Impatiens species reconstructed the taxonomic relationship and provided detailed information about nucleotide diversity hotspots, which could facilitate the systematic evolution research of the Balsaminaceae species [23]. Furthermore, various chloroplast genome regions and DNA barcodes have been considered as useful molecular markers in systematic and population genetic studies to distinguish its closely related species and adulterants [24]. For instance, four chloroplast single nucleotide polymorphism (SNP) variants were identified as powerful markers to differentiate rubber dandelion species from weedy relatives [25]. The DNA barcodes *rps16* and *trnQ-UUG* were designed based on the comparative analysis of complete chloroplast genome sequences of *Conyza bonariensis*, which could successfully separate three predominant Conyza species [26]. More importantly, DNA barcodes from chloroplast genome exhibited the potential as a powerful analytical tool for determination of geographical origins. The haplotype map generated by four chloroplast DNA markers identified 10 informative intra specifically variable sites, providing useful molecular tool for tracking Merbau timber originating from peninsular Malaysia [27]. The cp DNA marker *trnL-F* was successfully applied to investigate phylogeographical pattern of 27 populations of *Chrysanthemum indicum* across the southwest to northwest areas in China, which revealed fifteen haplotypes and correlative high genetic differentiation among populations [28]. The DNA barcodes from chloroplast genomes would be promising approach to distinguish the geographical origins of *T. hemsleyanum*, as well as rapidly differentiate the genuine and adulterated crude drugs. Therefore, it is highly important and essential to investigate the complete chloroplast genomes of *T. hemsleyanum* from different regions and develop typical molecular markers to discriminate their provenances for its further clinical application and development.

In the current study, we have sequenced and assembled the cp genomes of *T. hemsleyanum* collected from five different regions using Illumina sequencing platform. These sequences were further compared with other known chloroplast genomes from Vitaceae species to reveal the conserved and different features on basic genome structure, codon usage bias, repetitive structure characteristics and IR expansion. The phylogenetic relationship between *Tetrastigma* species and other closely related taxa from Vitaceae were reconstructed to infer the taxonomic status of *Tetrastigma* climbers within the families. Finally, we designed two specific DNA barcodes to identify the geographical origins of *T. hemsleyanum*, which successfully divided the *T. hemsleyanum* climbers from different regions into four clades. The genomic and marker resources described here expanded our understanding of the diversity of chloroplast genomes and their taxonomic relationships within Vitaceae species, and provided an efficient molecular approach for the geographical origin identification of *T. hemsleyanum*.

## Results

### Morphological features and chloroplast genome characteristics of Tetrastigma hemsleyanum

**Fig. 1:**
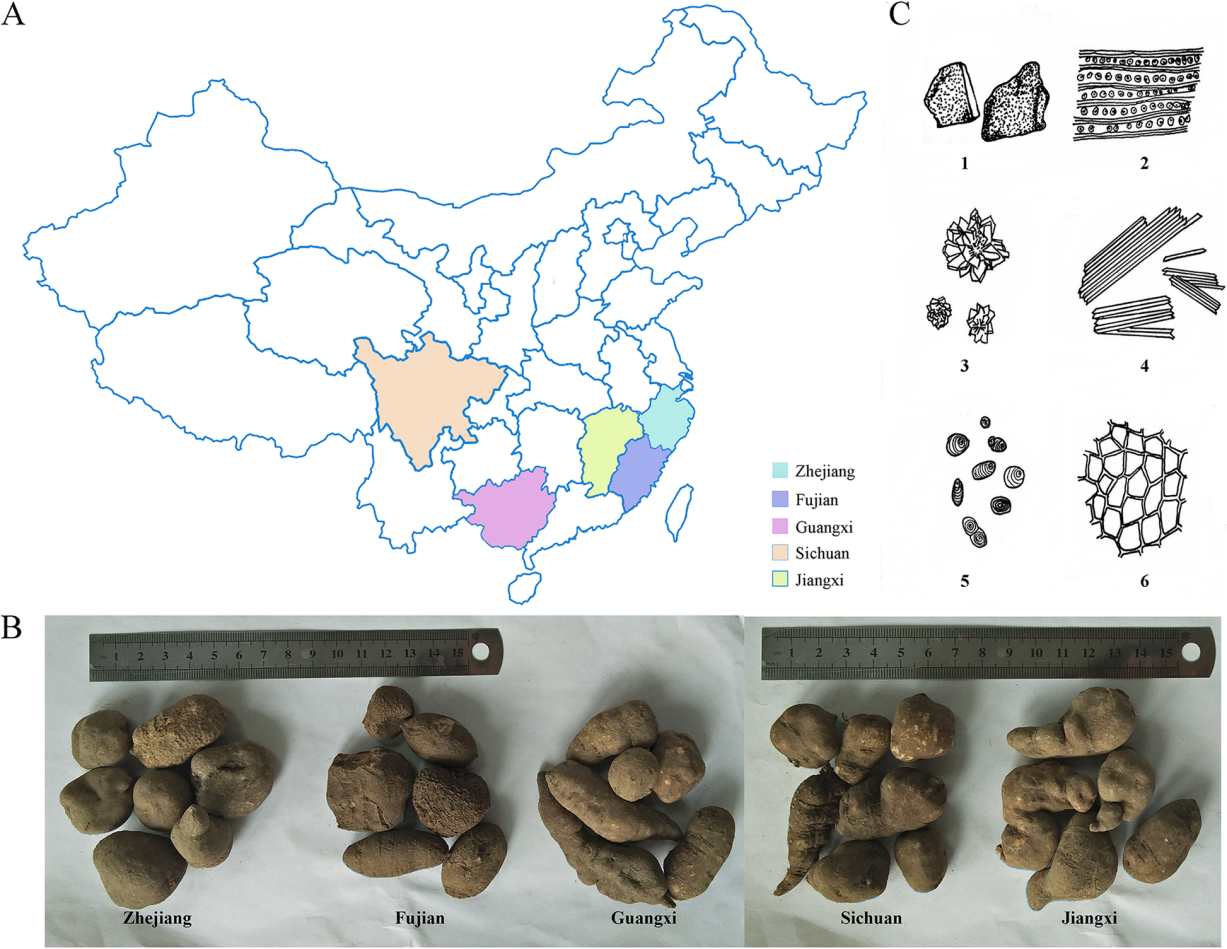
The geographical distributions and morphological characteristics of *Tetrastigma hemsleyanum* from different regions. **A** Regional distribution map of *Tetrastigma hemsleyanum* sample collection (**B**) The microscopic characters of the dried roots from *Tetrastigma hemsleyanum*: 1. brown patches; 2. marginal orifice catheter; 3. cluster crystals of calcium oxalate; 4. needle crystals of calcium oxalate; 5. starch granules; 6. cork cell. **C** Comparison of macroscopic characters of the root tubers of *Tetrastigma hemsleyanum* from five different regions

The samples of *Tetrastigma hemsleyanum* were collected from Zhejiang, Fujian, Jiangxi, Guangxi and Sichuan provinces, which have been indicated as the main producing zones of *T. hemsleyanum* crude drugs (Fig. 1A). The external morphology and microstructure of the root tubers of *T. hemsleyanum* from different regions were analyzed to identify the differences on morphological features. As shown in Fig. 1B, all of the root tubers from different regions exhibited similar morphological characters, including the root tuber size, the shape of elliptical or spindle, and the epidermis with tan. Moreover, most of root tubers of *T. hemsleyanum* showed smooth appearance, while a few of them presented folds and lenticel-like protuberances, as well as depressions (Fig. 1B). In addition, the microscopic features of the powder revealed that the cork cells, brown patches, needle crystals of calcium oxalate, starch granules and marginal orifice catheters were abundant in root tubers of *T. hemsleyanum* from five different regions, while cluster crystals of calcium oxalate were rarely observed in the tubers (Fig. 1C). However, the pharmacognostical analysis failed to identify significant differences on external morphology and microstructure of root tubers of *T. hemsleyanum* from different regions, requiring the development of alternative strategies to discriminate its geographical origin. Therefore, we sequenced the complete cp genome of *T. hemsleyanum* from five different regions and conducted a comparative analysis to establish and develop potential molecular approach for geographical origins traceability of *T. hemsleyanum*.

**Fig. 2:**
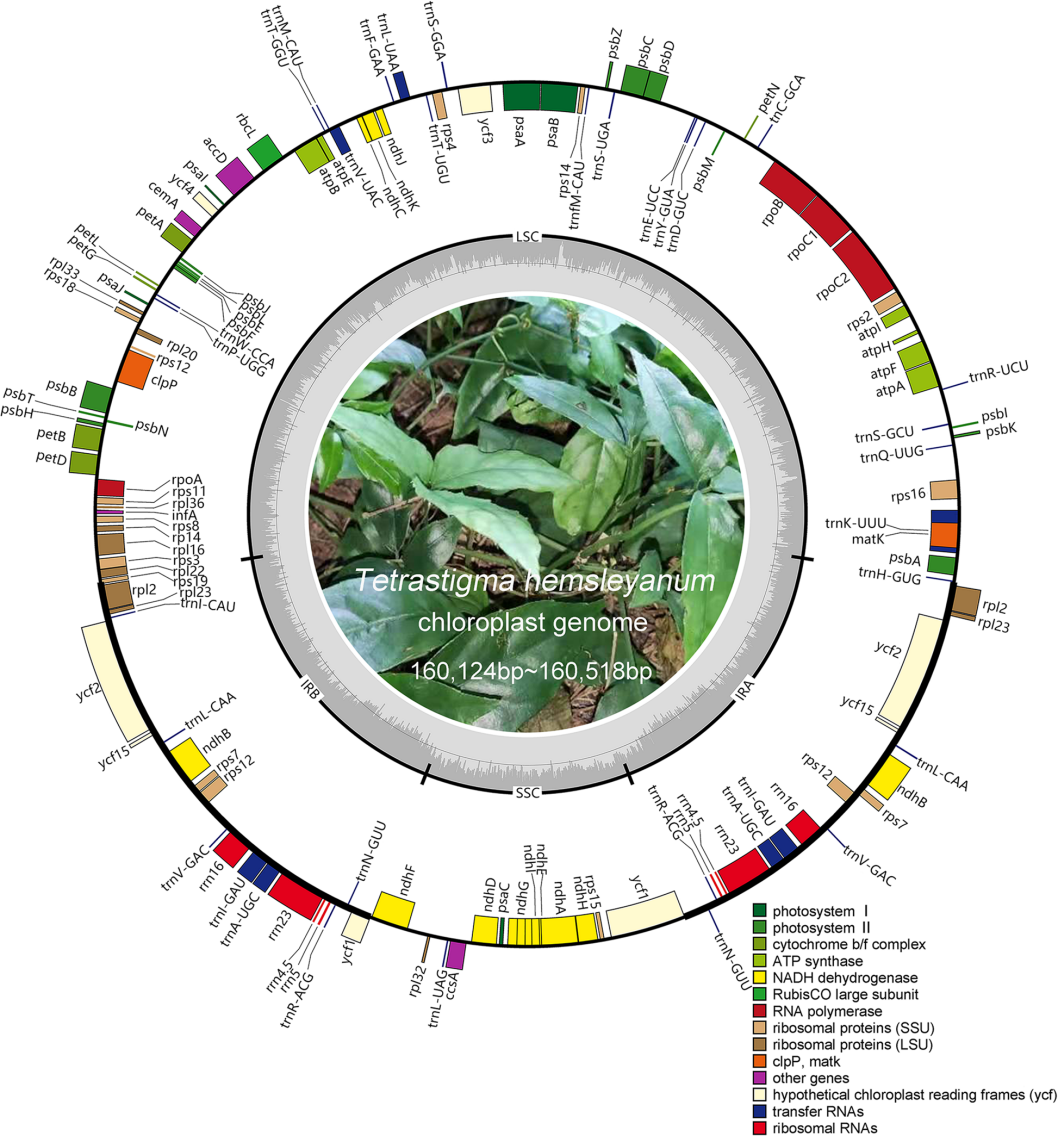
The chloroplast genome map of *Tetrastigma hemsleyanum* from five different regions. Genes labeled inside the circle are transcribed clockwise, while those outside the circle are transcribed anti-clockwise. The tick lines exhibited the extent of the Inverted Repeats (IRA and IRB) separating the Large Single-Copy (LSC) and the Small Single-Copy (SSC) regions. The darker gray and the lighter gray in the inner circle corresponds to GC and AT content, respectively. Genes with different functions are represented in different colors

The complete chloroplast sequences of *T. hemsleyanum* from five different regions of Zhejiang, Fujian, Guangxi, Sichuan and Jiangxi Provinces, have been deposited in the GenBank database with the accession No. of MW375707 ~ MW375711. The size of the whole chloroplast genomes of *T. hemsleyanum* varied from 160,124 bp to 160,518 bp, with the smallest and largest *T. hemsleyanum* cp genome from Jiangxi and Zhejiang Province, respectively. All of the five *T. hemsleyanum* cp genomes exhibited a typical angiosperm circular chloroplast structure containing four regions: large single-copy region (LSC; 88,131 bp-89,298 bp), small single-copy region (SSC; 18,962 bp-18,965 bp), and a pair of inverted repeats (IR; 26,126 bp-26,517 bp) (Fig. 2). A total of 112 genes, including 80 protein-coding genes, 28 tRNAs, and 4 rRNAs were identified from each genome of *T. hemsleyanum* from different regions (Table 1). The cp genomes showed high similarity in terms of gene contents, orders and orientations. Specifically, the overall GC contents of *T. hemsleyanum* from five regions revealed almost the same results in five regions, among which medicinal plant from Zhejiang, Fujian and Guangxi exhibited a GC content of 37.50%, while that from other two regions showed a result of 37.52% (Table 2). No significant differences on protein coding genes were identified in the *T. hemsleyanum* cp genomes from different regions, with a total length of 80,022 bp. There were 18 duplicated genes identified in the IR regions of *T. hemsleyanum* cp genome including 8 protein coding genes (*rpl2*, *rpl23*, *ycf1*, *ycf2*, *ycf15*, *ndhB*, *rps12* and *rps7*), 7 tRNA genes (*trnA-UGC*, *trnI-CAU*, *trnI-GAU*, *trnL-CAA*, *trnN-GUU*, *trnR-ACG* and *trnV-GAC*) and 4 rRNA genes (*rrn4.5*, *rrn5*, *rrn16*, *rrn23*). (Table 2). Furthermore, 18 distinct genes were indicated as intron-containing genes in the cp genome of *T. hemsleyanum*, including 13 protein coding genes and 5 tRNA genes. All these genes exhibited a single intron, except for *rps12*, *clpP* and *ycf3* which contained two introns. Moreover, it is intriguing that the location and the intron aera of *rpl2* gene were diverse in *T. hemsleyanum* cp genomes from different genomes. The *rpl2* gene of Guanxi and Zhejiang *T. hemsleyanum* cp genomes possessed two introns and across the junction of IRA and LSC region, which occupied in LSC region with 149 bp and 223 bp respectively. While the *rpl2* gene of *T. hemsleyanum* cp genome from other three regions showed only one intron and located in IRA completely. The above results indicated the cp genomes of *T. hemsleyanum* from different regions were slightly different, but it is highly conserved on basic structure, genome size, gene number and total GC content.

**Table 1:** List of genes annotated in the chloroplast genomes of *Tetrastigma hemsleyanum*

Classification of genes	Gene Names	Number
Photosystem I	*psaA, psaB, psaC, psaI, psaJ*	5
Photosystem II	*psbA, psbB, psbC, psbD, psbE,psbF, psbH, psbI, psbJ, psbK, psbL, psbM, psbN, psbT, psbZ*	15
Cytochrome b/f complex	*petA, petB, petD, petG, petL, petN*	6
ATP synthase	*atpA, atpB, atpE, atpF, atpH, atpI*	6
NADH dehydrogenase	ndhA, ndhB*, ndhC, ndhD, ndhE, ndhF, ndhG, ndhH, ndhI, ndhJ, ndhK	11
RubisCO large subunit	*rbcL*	1
RNA polymerase	*rpoA, rpoB, rpoC1, rpoC2*	4
Ribosomal proteins (SSU)	rps2, rps3, rps4, rps7*, rps8, rps11, rps12*, rps14, rps15, rps16, rps18, rps19	12
Ribosomal proteins (LSU)	rpl2*, rpl14, rpl16, rpl20, rpl22, rpl23*, rpl32, rpl33, rpl36	9
Ribosomal RNAs	rrn 4.5*，rrn 5*, rrn 16*, rrn 23*	4
Protein of unknown function	ycf1*, ycf2*, ycf3, ycf4, ycf15*	5
Transfer RNAs	trnA-UGC*, trnC-GCA, trnD-GUC, trnE-UUC, trnF-GAA, trnH-GUG, trnI-CAU*, trnI-GAU*, trnK-UUU, trnL-CAA*, trnL-UAA, trnL-UAG, trnfM-CAU, trnM-CAU, trnN-GUU*, trnP-UGG, trnQ-UUG, trnR-ACG*, trnR-UCU, trnS-GCU, trnS-GGA, trnS-UGA, trnT-GGU, trnT-UGU, trnV-GAC*, trnV-UAC, trnW-CCA, trnY-GUA	28
Other genes	*accD, ccsA, cemA, clpP, infA, matK*	6
Total		112

*indicate a duplicated gene

**Table 2:** Statistics on the basic feature of the cp genomes of five *T. hemsleyanum* plants and three Vitaceae species

Characteristics	***Tetrastigma hemsleyanum***					***Tetrastigma planicaule***	***Ampelopsis japonica***	***Vitis vinifera***
	Zhejiang	Fujian	Guangxi	Sichuan	Jiangxi			
Genbank accession No.	MW375707	MW375708	MW375709	MW375710	MW375711	MW401672	NC_042235	NC_007957
Total length (bp)	160,518	160,152	160,153	160,127	160,124	160,323	161,430	160,928
LSC length (bp)	89,298	88,183	88,798	88,131	88,142	88,181	89,626	89,147
SSC length (bp)	18,968	18,965	18,965	18,962	18,962	19,096	18,977	19,065
IR length (bp)	26,126	26,502	26,195	26,517	26,510	26,523	26,413	26,358
Gene number (bp)	112	112	112	112	112	113	114	113
Gene number in IR regions	19	19	19	19	19	18	19	18
Protein-coding gene number	80	80	80	80	80	79	80	79
rRNA gene number	4	4	4	4	4	4	4	4
tRNA gene number	28	28	28	28	28	30	30	30
Total GC content (%)	37.50	37.50	37.50	37.52	37.52	37.49	37.32	37.40

To further determine the conserved and variable structures of cp genome in family Vitaceae, we conducted a comparative analysis between *T. hemsleyanum* plant and other species from tribe Cayratieae (*Tetrastigma planicaule*), tribe Ampelopsideae (*Ampelopsis japonica*) and tribe Viteae (*Vitis vinifera*). The structure of the chloroplast genome appeared to be largely conserved across the family Vitaceae, with little differences on the total genome length, gene number and GC content (Table 2). The size of the chloroplast genome varied from 160,323 bp in *T. planicaule* to 161,430 bp in *A. japonica*, and the overall GC content ranged from 37.32% (*A. japonica*) to 37.49% (*T. planicaule*). However, the types and numbers of genes coded in the cp genomes of *T. planicaule*, *A. japonica* and *V. vinifera* were not identical with that of *T. hemsleyanum*. The lack of *ycf15* gene resulted in a decrease in the number of protein coding genes of *T. planicaule* and *V. vinifera*, while the protein coding gene number of *A. japonica* was consistent with that of *T. hemsleyanum*. In addition, the *ycf1* gene of *V. vinifera* completely located in IRB region and was indicated as a pseudogene copy. Compared with the protein coding genes, more significant differences were identified on the the tRNA genes among the cp genomes from the four Vitaceae plants. The *trnS-GCU* gene was uniquely encoded by *T. hemsleyanum*, while *trnG-GCC*, *trnG-UCC*, *trnV-GAU* were solely encoded by other three Vitaceae species, which led to the uniqueness of *T. hemsleyanum*.

### Comparative analyses of chloroplast genome

As a link between the nucleic acids and proteins, the genetic code plays an important role in the transmission of genetic information in organisms [29]. Therefore, we analyzed the codon distribution among the protein coding genes in cp genome of *T. hemsleyanum* from different regions and performed a comparison analysis. The cp genomes of *T. hemsleyanum* from five regions exhibited almost identical protein-coding sequences, which represented a total of 26,674 codons. All of these codons belonged to 64 codon types and encoded 20 amino acids (Supplementary Fig. 1). However, the numbers of amino acid and the bias of codon usage of *T. hemsleyanum* cp genomes from different regions exhibited a slight disparity. Leucine was the most abundant amino acid (2774 ~ 2776 codons, 10.40% ~ 10.41% of the total), whereas Cysteine (320 ~ 322 codons, 1.20% ~ 1.21% of the total) showed the least abundance in the cp genome of *T. hemsleyanum*. Regardless of stop codons, the most commonly applied codon was AUU (1117 ~ 1118), encoding isoleucine and the least one was UGC (89 ~ 91), encoding cysteine (Supplementary Table 3). The single most striking observation to emerge from the data comparison in Supplementary Table 3 was that the codon usage patterns of *T. hemsleyanum* from five different regions could be divided into three types. According to the data in Supplementary Table 3, the codon usage bias of *T. hemsleyanum* from Jiangxi and Sichuan were exactly the same, while those from Fujian and Guangxi completely displayed the same bias, and the special one from Zhejiang exhibited a unique pattern of codon usage differing from the other regions. Further comparative analysis revealed that a total of 28 variants sites in 21 protein-coding genes of *T. hemsleyanum* from different regions, which led to discrepancies in codon usage preference and the number of amino acid coding (Supplementary Table 4). What stands out in the Supplementary Table 4 was that, the protein coding genes of *atpB*, *ccsA*, *ycf2* and *ycf1* exhibited two variable sites while *accD* gene displayed 3 mutation sites. The more surprising correlation was the variant sites of *accD* gene in *T. hemsleyanum* from Jiangxi and Sichuan provinces resulted in the encoding of lysine, which was obviously distinguished with methionine, glutarnine and asparagine encoded in cp genome from other three regions. Comparing with *T. hemsleyanum* of Sichuan, Jiangxi and Zhejiang regions, one base variation was identified in the *ndhD* and *ycf2* genes of that from Fujian and Guangxi regions, which led to the preference of GGG and GGC, respectively (Supplementary Table 4). Taken together, these results provided important insights into the understanding of protein adaptive evolution and strategy development of identification geographical origin of *T. hemsleyanum*.

Previous reports have indicated that codon usage bias of chloroplast genome may be affected by selection, mutation and random drift [30, 31]. Further comparing analysis between *T. hemsleyanum* and other three Vitaceae species suggested the coded amino acids of *T. planicaule*, *A. japonica* and *V. vinifera* were identical with that of *T. hemsleyanum*. The numbers of codons in the cp genome of *T. planicaule*, *A. japonica* and *V. vinifera* were 26,978, 26,990 and 26,124, respectively (Supplementary Table 3). On average, the most abundant amino acids in the three species were leucine (*T. planicaule* 2800, 10.38%; *A. japonica* 2724, 10.09%; *V. vinifera* 2803, 10.73%) whereas the least abundant amino acid was Cysteine (*T. planicaule* 327, 1.21%; *A. japonica* 308, 1.14%; *V. vinifera* 325, 1.24%). In addition, similar codon usage patterns were observed among the eight Vitaceae plants. As shown in Supplementary Table 3, most of the amino acid showed codon preferences in the cp genomes of Vitaceae plants. However, methionine (AUG) and tryptophan (UGG) were encoded by only one codon and exhibited no codon preferences. AGA (1.87 ~ 1.90) in arginine showed the highest RSCU value, and the lowest one was AGC (0.34 ~ 0.36) in serine. Moreover, the RSCU value for each Vitaceae species exhibited similar codon preference in the 64 codons in the CDS genes. As a result, 31 of them for each species exhibited greater preference (RSCU > 1), indicating an obvious codon bias in the amino acids. Most (29 codons) of these preferred codons among eight Vitaceae plants species ended with the nucleotide of A or U. Therefore, the investigation on codon preferences is conducive to understand the exogenous gene expression and the molecular evolution mechanisms of *T. hemsleyanum* in Vitaceae.

**Fig. 3:**
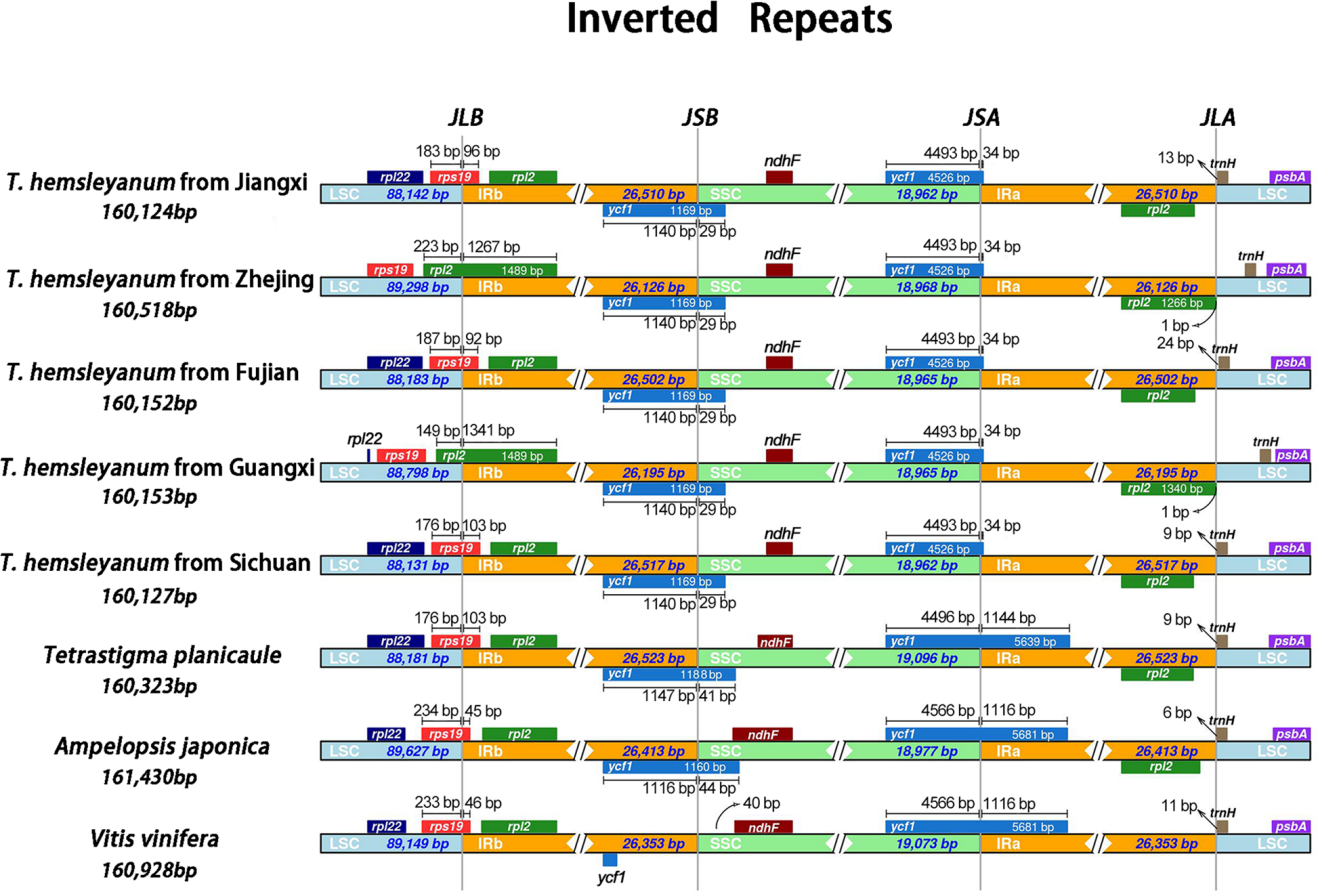
Comparison of the LSC, IR, and SSC junction regions among five *Tetrastigma hemsleyanum* samples with different geographical origins, *Tetrastigma planicaule*, *Ampelopsis japonica* and *Vitis vinifera* cp genomes

Contraction and expansion of the IR region is a common phenomenon known as ebb and flow, which could be used as effective tool for phylogenetic relationship and classification research of medicinal plants [32]. A comparison of five *T. hemsleyanum* plants and three Vitaceae species for borders was performed between the IRs and two single copies regions in detail. The length of the IR regions was similar among the eight Vitaceae species ranging from 26,126 bp in *T. hemsleyanum* (Zhejiang) to 26,523 bp in *T. planicaule*, with certain expansion and contraction (Fig. 3). Particularly, some notable differences were found at the boundaries among cp genomes of *T. hemsleyanum* from different regions. The LSC-IRb border was located within the *rps19* gene in *T. hemsleyanum* from Jiangxi, Fujian and Sichuan province, with extending length of 96 bp, 92 bp and 103 bp to the IRB region, respectively (Fig. 3). However, the *rps19* gene was completely encoded in LSC region and exhibited 290 and 216 bp distance to the junction of the LSC-IRB region in the cp genome of *T. hemsleyanum* from Zhejiang and Guangxi, respectively (Fig. 3). In contrast, the locating position of *ycf1* gene was highly conserved at the boundary of IR/SSC region among Vitaceae plants except that of *V. vinifera*, which exhibited a pseudogene gene with the size of 1030 bp completely locating in IRB region (Fig. 3). The contraction and expansion of *ycf1* gene at the boundary of LSC-IRB within five *T. hemsleyanum* plants were identical, which occupied 1140 bp and 29 bp in IRB and SSC regions, respectively. Another interesting observation is that the overlap between *ycf1* gene and the IRA region was significantly longer of other three species than that of *T. hemsleyanum*, which showed 1144 bp for *T. planicaule* and 1116 bp for *A. japonica* and *V. vinifera*, respectively. However, the overlap length was only 34 bp in the cp genome of *T. hemsleyanum*, which could be considered to be one of the reasons for the length change among these Vitaceae cp genomes.

### Repeat sequences analysis and RNA editing sites identification

Long repeats are significant genetic resources, playing a crucial role in genome rearrangement and intermolecular recombination [33]. As shown in Supplementary Fig. 2, the long repeat sequences detected in *T. hemsleyanum* cp genomes of Jiangxi and Sichuan revealed the identical results, while specimens from Zhejiang, Fuajin and Guangxi regions exhibited slightly different types and number of repeat sequences. Within the five *T. hemsleyanum* plants, the long repeats analysis revealed the most abundant repeats were length of 30–39, with the largest number in type of forward repeats (27–28), followed by palindrome (18–19), complemented (2) and reverse (2) repeats. These results further confirmed the high similarities on the type of repeats and certain slight variations on terms of the number and length among cp genomes of *T. hemsleyanum* from different regions (Supplementary Fig. 2A). However, the comparison of *T. hemsleyanum* with the other three Vitaceae species displayed an obvious distinction. A total of 49, 48, 40 long repeats were identified in the cp genomes of *T. planicaule*, *A. japonica* and *V. vinifera* respectively. In contrast with the cp genome of *T. hemsleyanum*, no complemented repeats were determined in the cp genome of other three Vitaceae plants. In addition, the type of reverse repeats was also lost in the cp genome of *T. planicaule*. Among these Vitaceae plants, most of the repeat units were short, ranging from 30 to 59 bp (Supplementary Fig. 2B).

**Fig. 4:**
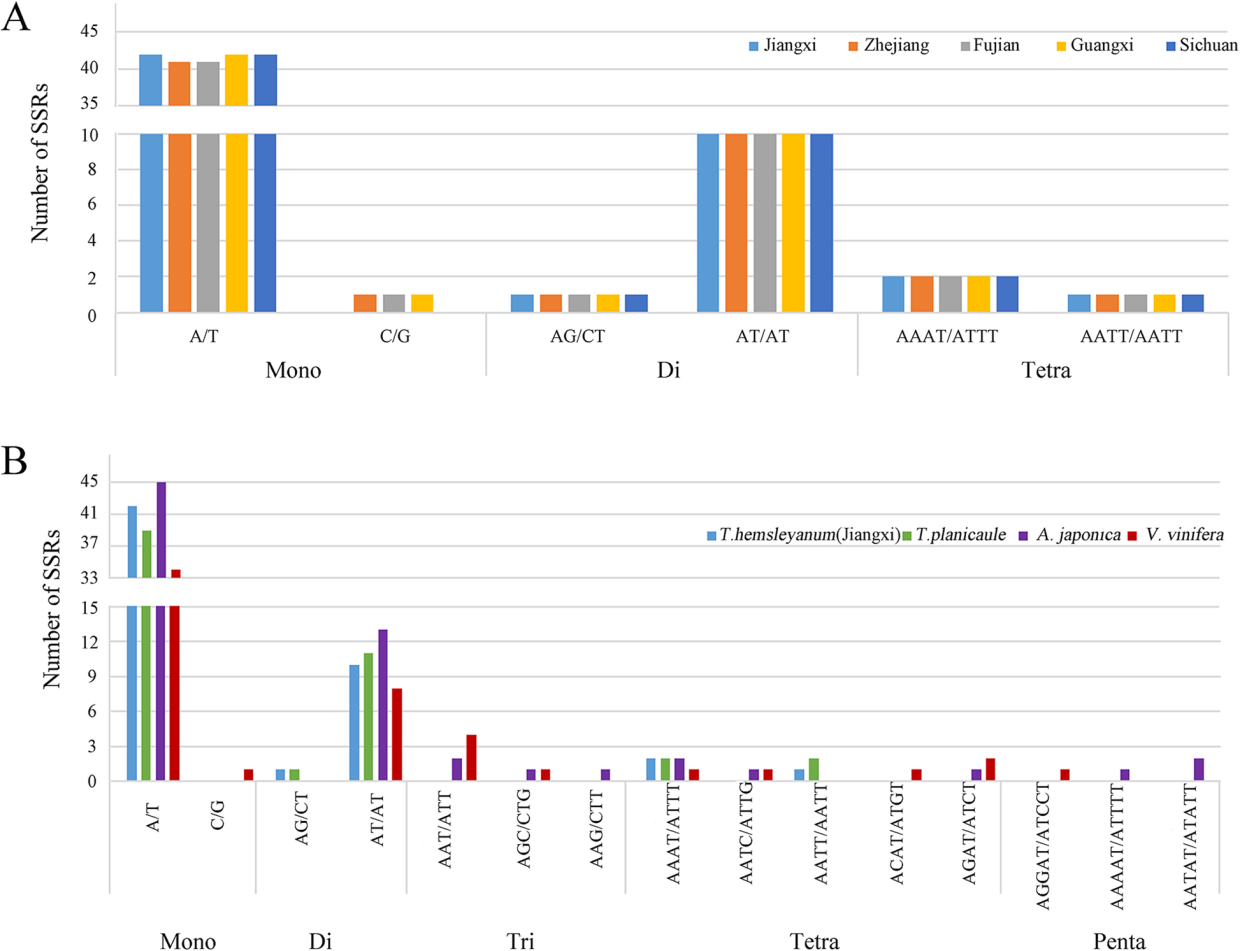
Analysis of simple sequence repeats (SSRs) in Vitaceae plants and *Tetrastigma hemsleyanum* species with different geographical origins. **A** The number of different types of SSRs in five samples of *Tetrastigma hemsleyanum* from different regions. **B** The number of different types of SSRs in the cp genomes of *Tetrastigma hemsleyanum* sample from Jiangxi Province, *Tetrastigma planicaule*, *Ampelopsis japonica* and *Vitis vinifera*

Simple sequence repeats (SSRs) play an essential role in plant taxonomy and population genetics studies for the high polymorphism and codominance [34]. In total, 56 SSRs were identified in the cp genomes of *T. hemsleyanum* plants from four regions, while the species from Guangxi exhibited a SSR number of 57. The majority of SSR sequences were mononucleotide repeats (42–43), followed by dinucleotides (11) and tetranucleotides (3) (Table 3). The cp genome of *T. hemsleyanum* of Jiangxi and Sichuan exhibited the identical results on SSR types and numbers. However, the distinctions of SSRs in *T. hemsleyanum* cp genomes from the other regions were embodied in SSRs count of mononucleotide repeats (Fig. 4A). Specifically, the numbers of A/T repeats in the cp genomes of *T. hemsleyanum* plants from Jiangxi, Zhejiang, Fujian, Guangxi and Sichuan were 42, 41, 41, 42 and 42, respectively. In addition, the samples from Jiangxi and Sichuan showed no C/G SSR repeats in the cp genomes. These results further indicated that SSR might be a useful molecular marker for species determination of geographical origins of *T. hemsleyanum*. In addition, a comparative SSRs analysis conducted with three Vitaceae species revealed 55, 69 and 54 SSRs were detected in the cp genomes of *T. planicaule*, *A. japonica* and *V. vinifera*, respectively (Table 3). It is must mentioned that *T. planicaule* from *Tetrastigma* genus showed identical SSRs types with slight distinctions on SSR quantities (Fig. 4B). Comparing with the *Tetrastigma* plants, *A. japonica* and *V. vinifera* possessed lots of additional types of SSRs and repeat nuits, containing mono-(45/35), di-(13/8), tri-(4/5), tetra-(4/5) and penta-(3/1) respectively. The extra SSR sequences include unique AAT/ATT, AGC/CTG, AAG/CTT, AATC/ATTG, AGAT/ATCT, AAAAT/ATTTT and AATAT/ATATT in *A. japonica* cp genome and AAT/ATT, AGC/CTG, AATC/ATTG, ACAT/ATGT, AGAT/ATCT and AGGAT/ATCCT in *V. vinifera* cp genome, respectively (Fig. 4B). Moreover, the lack of AG/CT and AATT/AATT in both of *A. japonica* and *V. vinifera* also revealed the discrepancy of SSR loci among different genus, which might also provide a basis for the identification of the *Tetrastigma* genus. Among all Vitaceae species, the number of SSRs composed by A/T were significantly greater than that containing G or C, indicating that the base composition of SSRs was biased toward the application of A/T bases, which was consistent with A-T enrichment in complete chloroplast genomes [35]. Taken together, these results provided important insights into understanding intrageneric and intergeneric variations within *T. hemsleyanum* and its relatives in Vitaceae species.

**Table 3:** The number and types of SSR in five *T. hemsleyanum* plants and three Vitaceae species

SSR type	Repeat unit	Amount							
		*T.h.* (Jiangxi)	*T.h.* (Zhejiang)	*T.h.* (Fujian)	*T.h.* (Guangxi)	*T.h.* (Sichuan)	*T. planicaule*	*A. japonica*	*V. vinifera*
Mono	A/T	42	41	41	42	42	39	45	34
	C/G	/	1	1	1	/	/	/	1
Di	AG/CT	1	1	1	1	1	1	/	/
	AT/AT	10	10	10	10	10	11	13	8
Tri	AAT/ATT	/	/	/	/	/	/	2	4
	AGC/CTG	/	/	/	/	/	/	1	1
	AAG/CTT	/	/	/	/	/	/	1	/
Tetra	AAAT/ATTT	2	2	2	2	2	2	2	1
	AATC/ATTG	/	/	/	/	/	/	1	1
	AATT/AATT	1	1	1	1	1	2	/	/
	ACAT/ATGT	/	/	/	/	/	/	/	1
	AGAT/ATCT	/	/	/	/	/	/	1	2
Penta	AGGAT/ATCCT	/	/	/	/	/	**/**	/	1
	AAAAT/ATTTT	/	/	/	/	/	**/**	1	/
	AATAT/ATATT	/	/	/	/	/	**/**	2	/
Total		56	56	56	57	56	55	69	54

The RNA editing process is an essential maturation mechanism to avoid incorrect RNA mutations and is widespread in the chloroplast genome of plants [36]. In total, 71 potential RNA editing sites have been predicted in 24 protein-coding genes of the cp genome of *T. hemsleyanum*, which displayed no distinction in numbers of RNA editing sites and conversions of amino acids in cp genome of *T. hemsleyanum* from different regions. (Table 4). Among the 71 RNA editing sites, 17 codons were observed to be edited at the first nucleotide position, whereas 54 codons were identified to be edited at the second nucleotide position, and no codons were edited at both of the first and second nucleotide. All of the identified codon changes in the cp genomes of *T. hemsleyanum* showed C to T conversions. Especially, the *ndhB* gene showed the largest number of RNA editing sites (11 editing sites), followed by *ndhD* (8 editing sites) and *ndhF* (7 editing sites), while nine genes (*accD*, *atpI*, *atpF*, *ccsA*, *clpP*, *psbE*, *psbF*, *psbL* and *rpl20*) exhibited only one editing site in *T. hemsleyanum* (Table 4). The RNA edition on protein gene resulted in a total of 11 kinds of amino acid conversions in the cp genome of *T. hemsleyanum*. The conversions of H to Y, L to F, P to S, R to W, R to C were due to codons edited at the first nucleotide position, while the S to L, P to L, S to F, T to M, A to V, T to I conversions were caused by codons edited at the second nucleotide position (Supplementary Table 5). The conversion of serine to leucine (S to L) was the most abundant kind of conversion, accounting for 42.3%, while arginine to tryptophan (R to W) and arginine to cysteine (R to C) were the least conversion, accounting for 1.4% merely (Supplementary Table 5). Furthermore, the predicted RNA editing sites in the cp genomes of *T. planicaule*, *A. japonica* and *V. vinifera* showed similar results with that of *T. hemsleyanum*, with the RNA editing sites number of 71, 72 and 70 respectively. The slight difference of the number of RNA editing sites were observed in *accD*, *ndhB* and *ndhF* genes among these Vitaceae plants, which led to the distinctions of amino acid conversions (Supplementary Table 5). Since the close correlation between RNA editing sites and nucleotide substitution of protein coding genes, we performed further analysis to investigate the synonymous substitutions (Ks) and non-synonymous substitutions (Ka) of protein coding genes with abundant RNA editing sites. The Ka/Ks ratios of most genes (22/24) in *T. hemsleyanum* were less than 0.5 expect the *matK* (0.5534) and *rps16* (0.5687), suggesting an obvious purifying selection pattern. Particularly, the *clpP*, *psbL* and *psbF* genes even exhibited a Ka/Ks value of 0, showing the three genes were possibly under strong purifying selection pressure (Table 5).

**Table 4:** Number of the RNA editing sites in the cp genome of *T. hemsleyanum* and three Vitaceae species

Gene	Number of RNA editing sites			
	*T. hemsleyanum*	*T.planicaule*	*A. japonica*	*V. vinifera*
*accD*	1	1	2	1
*atpA*	3	3	3	3
*atpF*	1	1	1	1
*atpI*	1	1	1	1
*ccsA*	1	1	1	1
*clpP*	1	1	1	1
*matK*	4	4	4	4
*ndhA*	4	4	4	4
*ndhB*	11	11	12	11
*ndhD*	8	8	8	8
*ndhF*	7	7	6	6
*ndhG*	3	3	3	3
*petB*	2	2	2	2
*psbE*	1	1	1	1
*psbF*	1	1	1	1
*psbL*	1	1	1	1
*rpl20*	1	1	1	1
*rpoA*	2	2	2	2
*rpoB*	5	5	5	5
*rpoC1*	2	2	2	2
*rpoC2*	4	4	4	4
*rps2*	2	2	2	2
*rps14*	2	2	2	2
*rps16*	3	3	3	3
Total	71	71	72	70

**Table 5:** The value of Ka/Ks in 25 protein coding genes with RNA editing sites in *T. hemsleyanum* (Jiangxi)

Gene	Number of RNA editing sites	non-synonymous substitutions (Ka)	synonymous substitutions (Ks)	Ka/Ks
*ndhB*	11	0.0113	0.0243	0.4650
*ndhD*	8	0.0516	0.3257	0.1584
*ndhF*	7	0.1087	0.3022	0.3597
*rpoB*	5	0.0240	0.2243	0.1070
*ndhA*	4	0.0383	0.2928	0.1308
*matK*	4	0.1296	0.2342	0.5534
*rpoC2*	4	0.0704	0.2663	0.2644
*ndhG*	3	0.0491	0.2718	0.1807
*atpA*	3	0.0334	0.2751	0.1214
*rps16*	3	0.1109	0.1950	0.5687
*rpoA*	2	0.0700	0.1879	0.3725
*rpoC1*	2	0.0267	0.2567	0.1040
*petB*	2	0.0124	0.1742	0.0712
*rps2*	2	0.0111	0.2074	0.0535
*rps14*	2	0.0263	0.1387	0.1896
*accd*	1	0.1093	0.2714	0.4027
*atpF*	1	0.0469	0.1480	0.3169
*atpI*	1	0.0263	0.1612	0.1631
*ccsA*	1	0.0844	0.2810	0.3004
*clpP*	1	0	0	0
*psbE*	1	0.0053	0.2050	0.0259
*psbF*	1	0	0.0934	0
*psbL*	1	0	0	0
*rpl20*	1	0.0709	0.1643	0.4315

### Phylogenetic analysis

**Fig. 5:**
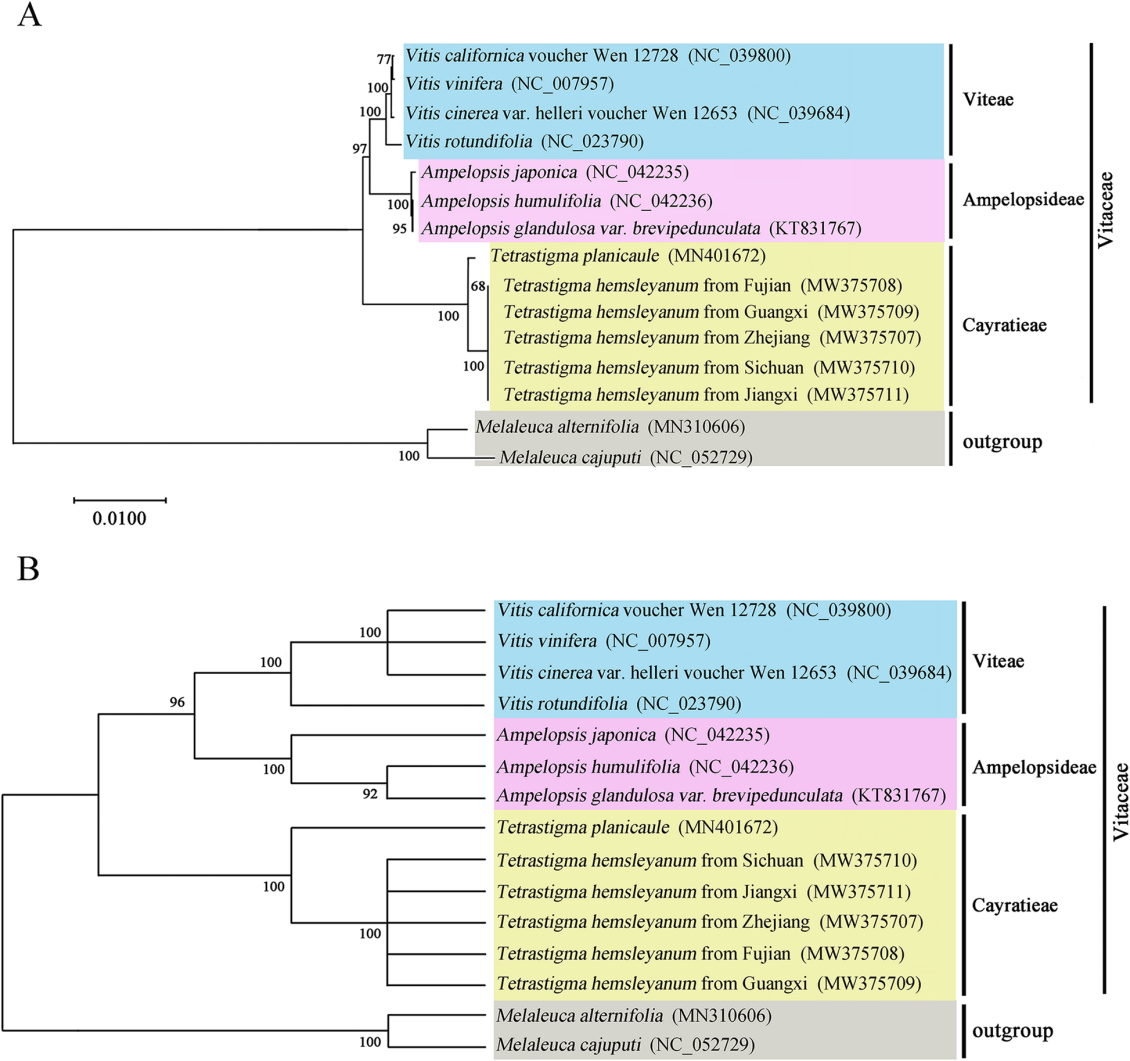
Phylogenetic relationships based on the conserved chloroplast protein coding genes from five samples of *Tetrastigma hemsleyanum* and other representative Vitaceae species. **A** The tree was constructed using maximum likelihood (ML) method. **B** The tree was constructed using maximum parsimony (MP) method. The number above each node referred to the bootstrap value from 500 replicates. The areas corresponding to blue, lavender and yellow represented the tribes of Viteae, Ampelopsideae and Cayratieae, respectively. *Melaleuca alternifolia* and *Melaleuca cajuputi* were set as the outgroups for phylogenetic analysis

The previous reports by molecular and morphological data indicated the family of Vitaceae could be classified into five major clades, including the tribe of Ampelopsideae, Cisseae, Cayratieae, Parthenocisseae, and Viteae [37]. However, the deep phylogenetic relationship of Vitaceae still needs further explorations to reveal the evolutionary characters and genetic status of grape species. Therefore, we constructed phylogenetic tree of Viteae family based on the 70 protein-coding gene datasets by maximum likelihood (ML) and maximum parsimony (MP) method, respectively. These grape plants contained 4 species from tribe Viteae, 3 species from tribe Ampelopsideae and 6 plants from tribe Cayratieae. *Melaleuca alternifolia* and *M. cajuputi* were chosen as the outgroups for phylogenetic analysis. As shown in Fig. 5, nearly all of the nodes received moderate to high support values in the ML and MP tree analysis. However, several topological differences have been occurred in relationships within the five *T. hemsleyanum* species and the tribe of Viteae between the ML and MP tree results (Fig. 5). The phylogenetic analysis among the five *T. hemsleyanum* plants revealed a stable monophyletic group with high bootstrap values, which exhibited a stable sister relationship with *T. planicaule*, indicating a close genetic relationship within the genus of *Tetrastigma* (Fig. 5). In addition, the ML analysis indicated that the samples of *T. hemsleyanum* from Fujian and Guangxi regions clustered together to form a combined group with a bootstrap score of 68, which subsequently gathered together with *T. hemsleyanum* species from other three regions (Fig. 5A).

These results indicated certain subtle protein coding differences of *T. hemsleyanum* cp genomes from different regions, providing potential molecular tools for distinguishing the geographical origins of *T. hemsleyanum*. Furthermore, the Ampelopsideae species and Viteae plants combined together to form a clade with strong statistical support, which combined with six *Tetrastigma* plants to form a robust monophyletic clade, which was consist with the previous classification of the tribes of Ampelopsideae, Viteae and Cayratieae in Vitaceae.

### Nucleotide diversity analysis and development of molecular marker for geographical origin discrimination

**Fig. 6:**
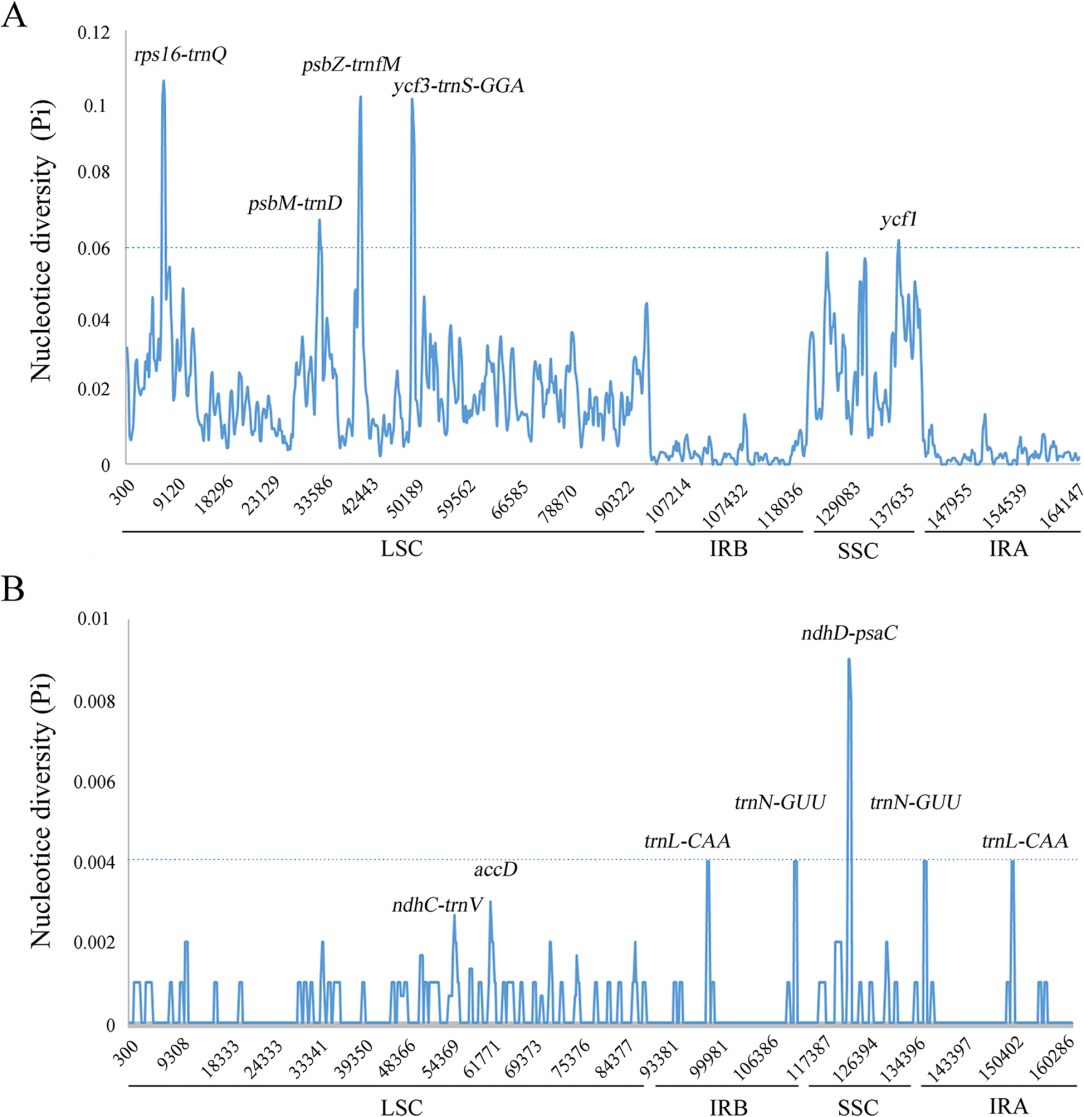
Comparison of potential mutational hotspots in the complete chloroplast genomes among Vitaceae plants and *Tetrastigma hemsleyanum* samples from different regions. **A** Nucleotide diversity (Pi) analysis among four Vitaceae chloroplast genomes. **B** Nucleotide diversity (Pi) analysis in the cp genomes of *Tetrastigma hemsleyanum* from five different regions. Sliding window length was 800 bp and step size was selected as 200 bp. The Pi value of each window is shown on the *Y*-axis, and their positions of the midpoint represented on the *X*-axis

The complete cp genomes with high variable levels provides potential molecular marker for species identification and geographical origin determination. In order to assess the sequences divergence level within the Vitaceae species, the complete cp genomes have been multiple aligned and applied DnaSP to calculate nucleotide variability (Pi). As shown in Fig. 6A, the sliding window analysis revealed 5 highly variable regions with Pi values ranging from 0.06194 to 0.10611 across four complete cp genomes of Vitaceae species, including 4 intergenic regions (*rps16-trnQ*, *psbM-trnD*, *psbZ-trnfM* and *ycf3-trnS*) and one protein coding genes (*ycf1*) (Fig. 6A). Among the five mutational hotspot loci, four highly variable hotspots were located in the LSC region, and *ycf1* gene with the Pi value of 0.06194 was identified in the SSC region. However, none of the hypervariable loci were determined in the IR region, further confirmed that the IR regions were highly conserved in the cp genomes among the Vitaceae species. The *rps16-trnQ* gene exhibited the highest Pi value of 0.10611, followed by *psbZ-trnfM* and *ycf3-trnS* with the Pi values of 0.10083 and 0.10056, respectively. Besides, a comparative analysis was carried out to determine the numbers of SNP sites and Gaps to further explore the characteristics of five hypervariable regions among four Vitaceae plants. The five mutational hotspots in the cp genome of *T. hemsleyanum* from Zhejiang province ranged from 892 bp (*psbZ-trnfM*) to 1139 bp (*rps16-trnQ*) in length (Table 6). Apparently, the high variable sequences of *T. planicaule* from *Tetrastigma* genus exhibited a small number of SNP sites (3–8) and Gaps (0–19) than that of *T. hemsleyanum* from Jiangxi Province except *psbM-trnD* region, which contained the SNP site and Gaps of 79 and 39 in the cp genome of *T. planicaule*, respectively. However, a great deal of variable sites was displayed in the 5 mutational hotspots of *A. japonica* and *V. vinifera*. For instance, the hypervariable regions of *psbZ-trnfM* showed 104 and 115 SNP sites in *A. japonica* and *V. vinifera*, respectively, which was significantly higher than that of *T. hemsleyanum* of Zhejiang. All these discrepancies led to variable mutational hotspot lengths in the Vitaceae plants eventually, and also provided potential molecular markers to resolve the difficulties in species identification of Vitaceae species.

**Table 6:** Multiple analysis of the mutational hotspots in four Vitaceae plants

mutational hotspots	Specises	Length	GC content	Number of SNP sites	Total length of Gaps
***rps16-trnQ***	*T. hemsleyanum* (Jiangxi)	1139 bp	23.09%	/	/
	*T. planicaule*	1141 bp	23.14%	5	4
	*A. japonica*	1208 bp	20.86%	149	167
	*V. vinifera*	1076 bp	21.84%	98	138
***psbM-trnD***	*T. hemsleyanum* (Jiangxi)	895 bp	35.08%	/	/
	*T. planicaule*	868 bp	35.37%	79	39
	*A. japonica*	860 bp	33.37%	60	77
	*V. vinifera*	843 bp	34.28%	41	100
***psbZ-trnfM***	*T. hemsleyanum* (Jiangxi)	892 bp	24.33%	/	/
	*T. planicaule*	883 bp	24.46%	8	19
	*A. japonica*	907 bp	24.33%	104	95
	*V. vinifera*	911 bp	22.39%	115	69
***ycf3-trnS***	*T. hemsleyanum* (Jiangxi)	1031 bp	33.56%	/	/
	*T. planicaule*	1029 bp	33.92%	3	2
	*A. japonica*	1113 bp	33.33%	97	128
	*V. vinifera*	1123 bp	33.93%	97	146
***ycf1***	*T. hemsleyanum* (Jiangxi)	977 bp	30.60%	/	/
	*T. planicaule*	977 bp	30.40%	6	0
	*A. japonica*	965 bp	29.95%	77	12
	*V. vinifera*	980 bp	30.61%	67	15

To determine the potential of variable sequences in cp genome for geographical origin discrimination, we further evaluate the the sequences divergence level of *T. hemsleyanum* from different regions. The results demonstrated that the intraspecific differences of *T. hemsleyanum* was much lower than interspecific differences among Vitaceae species (Fig. 6). A total of 5 mutational hotspots with relative high Pi value (≥0.004) have been screened out in *T. hemsleyanum* plants, including 2 hypervariable regions (*trnL-CAA* and *trnN-GUU*) located in IRs and one intergenic region located in SSC (*ndhD-psaC*) with the Pi value of 0.009 (Fig. 6B). Accordingly, these hypervariable regions with abundant intraspecific variable sites could be developed as potential DNA barcodes to discriminate the geographical origins of *T. hemsleyanum*. Interestingly, we found that both the SSC and IR regions were more variable than the LSC region in the chloroplast genomes of *T. hemsleyanum* from different regions. This result was significant different with the general observations in other species, where the IR regions usually exhibited lower variability than the LSC and SSC regions. One important reason was conjectured that the intraspecific variation among individuals was influenced by genes of NADH dehydrogenase mostly distributed in SSC region and transfer RNAs in IR regions, while the interspecific evolution among different species was driven by genes of Photosystem I/II, ATP synthase and Ribosomal proteins located in LSC. This result also indicated that more attention should be focused on the dissimilarity of SSC region in cp genomes for breeding of excellent species.

**Fig. 7:**
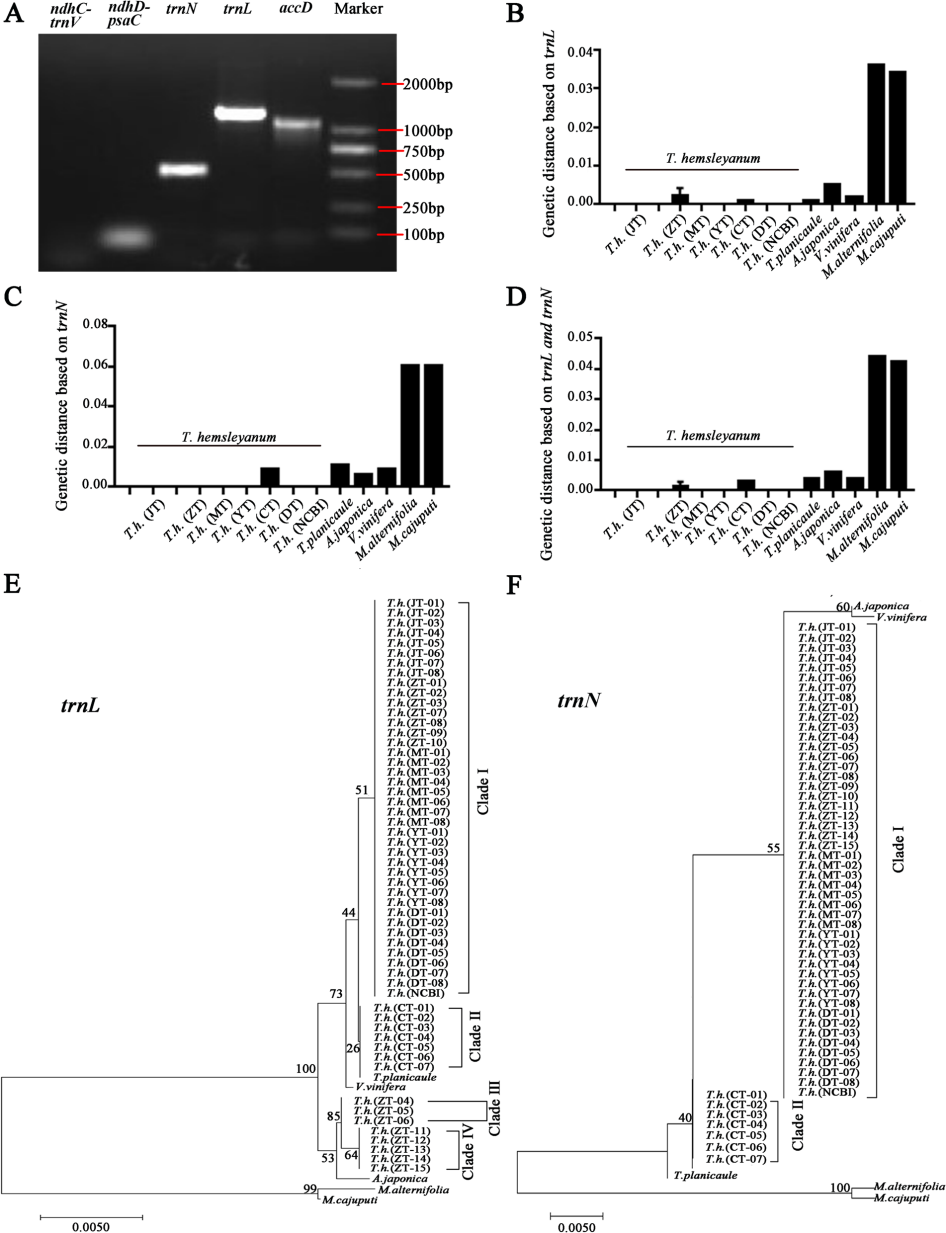
Comparative analysis of *trnL* and *trnN* sequences of *T. hemsleyanum* samples (**A**) Agarose gel electrophoresis of PCR products of five DNA barcodes from *T. hemleyanum* in Zhejiang Province. **B** Genetic distance analysis between the samples of *T. hemsleyanum* in Jiangxi Province and other regions, three representative Vitaceae species as well as two *Melaleuca* species based on *trnL* sequence, (**C**) *trnN* sequence and (**D**) combination of *trnL* + *trnN* sequences. The Neighbor-Joining (NJ) trees of 21 samples of *T. hemsleyanum* from different regions and 3 representative Vitaceae species, based on *trnL* sequence (**E**) and *trnN* sequence (**F**)

Our study designed five DNA barcodes (*accD*, *trnL-CAA*, *trnN-GUU*, *ndhD-psaC* and *ndhC-trnV*) based on hypervariable regions for PCR amplification of *T. hemsleyanum* medicinal materials in the Zhejiang region (Fig. 7A). The single bright band in agarose gel electrophoresis implied amplification of *accD*, *trnL-CAA* and *trnN-GUU*, while the *trnL-CAA* and *trnN-GUU* showed higher PCR amplification efficiency and sequence diversity. As a result, the two DNA barcodes were amplified with DNA of *T. hemsleyanum* samples from six different regions in batches to further analyze the efficiency of geographical origin discrimination. The detailed sequence information of the two PCR products is shown in Table 7. The size of the *trnL* and *trnN* barcodes in all *T. hemsleyanum* samples was 1143 bp and 469 bp, respectively. A total of six stable variants at position of 165 bp, 166 bp, 167 bp, 168 bp, 671 bp and 1036 bp were identified in the *trnL* sequence, generating four haplotypes of *T. hemsleyanum* from different regions (including our experiments and data from GenBank). The *trnL* sequences of *T. hemsleyanum* from Sichuan Province exhibited a unique haplotype of A4, while those from Zhejiang Province displayed three haplotypes of A1, A2 and A3 (Table 7). Notably, that *T. hemsleyanum* plants from Jiangxi, Zhejiang, Fujian, Guangxi and Guangdong regions harbored the identical *trnL* haplotype of A1, indicating the genetic variation of A1 was the main variety distributed in China due to its strong environmental adaptability. Additionally, the *trnN* sequences of *T. hemsleyanum* from different origins showed an identical GC content of 43.50%. However, these *trnN* sequences exhibited four variable bases at the position of 164 bp, 165 bp,166 bp and 167 bp, generating 2 haplotypes among different regions (Supplementary Table 6). Interestingly, the *trnN* sequence from Sichuan region showed a unique haplotype of B2, while that from other regions exhibited the same haplotype of B1 (Table 7). These results demonstrated that the intraspecies discrepancy of *T. hemsleyanum* plants among different regions, further confirming the availability and necessity of geographical origin identification strategy based on molecular markers of chloroplast genome.

**Table 7:** Sequence analysis of *T. hemsleyanum* samples and other Vitaceae species basing on two DNA barcodes

Species	Sample number	Sample source	***trnL-CAA***				***trnN-GUU***			
			Length	GC content	Genbank accession No.	Haplotype	Length	GC content	Genbank accession No.	Haplotype
*T. hemsleyanum*	JT-01	Lushan District, Jiujiang, Jiangxi 25°08′N, 117°02′E	1143 bp	38.85%	MZ995437	A1	469 bp	43.50%	MZ995468	B1
	JT-02				MZ995438	A1			MZ995469	B1
	JT-03				MZ995439	A1			MZ995470	B1
	JT-04				ON561859	A1			ON561826	B1
	JT-05				ON561860	A1			ON561827	B1
	JT-06				ON561861	A1			ON561828	B1
	JT-07				ON561862	A1			ON561829	B1
	JT-08				ON561863	A1			ON561830	B1
	ZT-01	Linhai, Taizhou, Zhejiang 28°85′N, 121°13′E			OK058531	A1			MZ995452	B1
	ZT-02				OK058532	A1			MZ995453	B1
	ZT-03				MZ995433	A1			MZ995454	B1
	ZT-07				ON561864	A1			ON561831	B1
	ZT-08				ON561865	A1			ON561832	B1
	ZT-09				ON561866	A1			ON561833	B1
	ZT-10				ON561867	A1			ON561834	B1
	ZT-04	Suichang County, Lishui, Zhejiang 28°61′N, 119°05′E			MZ995434	A2			MZ995455	B1
	ZT-05				MZ995435	A2			MZ995456	B1
	ZT-06				MZ995436	A2			MZ995457	B1
	ZT-11			38.93%	ON561868	A3			ON561835	B1
	ZT-12				ON561869	A3			ON561836	B1
	ZT-13				ON561870	A3			ON561837	B1
	ZT-14				ON561871	A3			ON561838	B1
	ZT-15				ON561872	A3			ON561839	B1
	MT-01	Longyan, Fujian 25°08′N, 117°02′E		38.85%	MZ995440	A1			MZ995458	B1
	MT-02				MZ995441	A1			MZ995459	B1
	MT-03				MZ995442	A1			MZ995460	B1
	MT-04				MZ995443	A1			MZ995461	B1
	MT-05				ON561873	A1			ON561840	B1
	MT-06				ON561874	A1			ON561841	B1
	MT-07				ON561875	A1			ON561842	B1
	MT-08				ON561876	A1			ON561843	B1
	YT-01	Baise, Guangxi 23°90′N, 106°62′E			MZ995444	A1			MZ995462	B1
	YT-02				MZ995445	A1			MZ995463	B1
	YT-03				MZ995446	A1			MZ995464	B1
	YT-04				ON561877	A1			ON561844	B1
	YT-05				ON561878	A1			ON561845	B1
	YT-06				ON624114	A1			ON561846	B1
	YT-07				ON561879	A1			ON561847	B1
	YT-08				ON561880	A1			ON561848	B1
	CT-01	Wanyuan, Dazhou, Sichuan 32°08′N, 108°03′E		38.76%	MZ995447	A4			MZ995465	B2
	CT-02				MZ995448	A4			MZ995466	B2
	CT-03				MZ995449	A4			MZ995467	B2
	CT-04				ON561881	A1			ON561849	B2
	CT-05				ON561882	A1			ON561850	B2
	CT-06				ON561883	A1			ON561851	B2
	CT-07				ON561884	A1			ON561852	B2
	DT-01	Shaoguan, Guangdong 24°80′N, 113°59′E		38.85%	MZ995450	A1			MZ995471	B1
	DT-02				MZ995451	A1			MZ995472	B1
	DT-03				ON561885	A1			ON561853	B1
	DT-04				ON561886	A1			ON561854	B1
	DT-05				ON561887	A1			ON561855	B1
	DT-06				ON561888	A1			ON561856	B1
	DT-07				ON561889	A1			ON561857	B1
	DT-08				ON561890	A1			ON561858	B1
	NCBI	Genbank			MT827073	A1			MT827073	B1
*T. planicaule*	TP-01			38.76%	MN401672	A5		43.28%	MN401672	B3
*A. japonica*	AJ-01		1136 bp	39.00%	NC_042235	A6			NC_042235	B4
*V. vinifera*	VV-01				NC_007957	A7		43.07%	NC_007957	B4
*M. alternifolia*	MA-01		1090 bp	38.53%	MN310606	A8	491 bp	41.75%	MN310606	B5
*M. cajuputi*	MC-01		1077 bp		NC_052729	A9		41.55%	NC_052729	B6

**Fig. 8:**
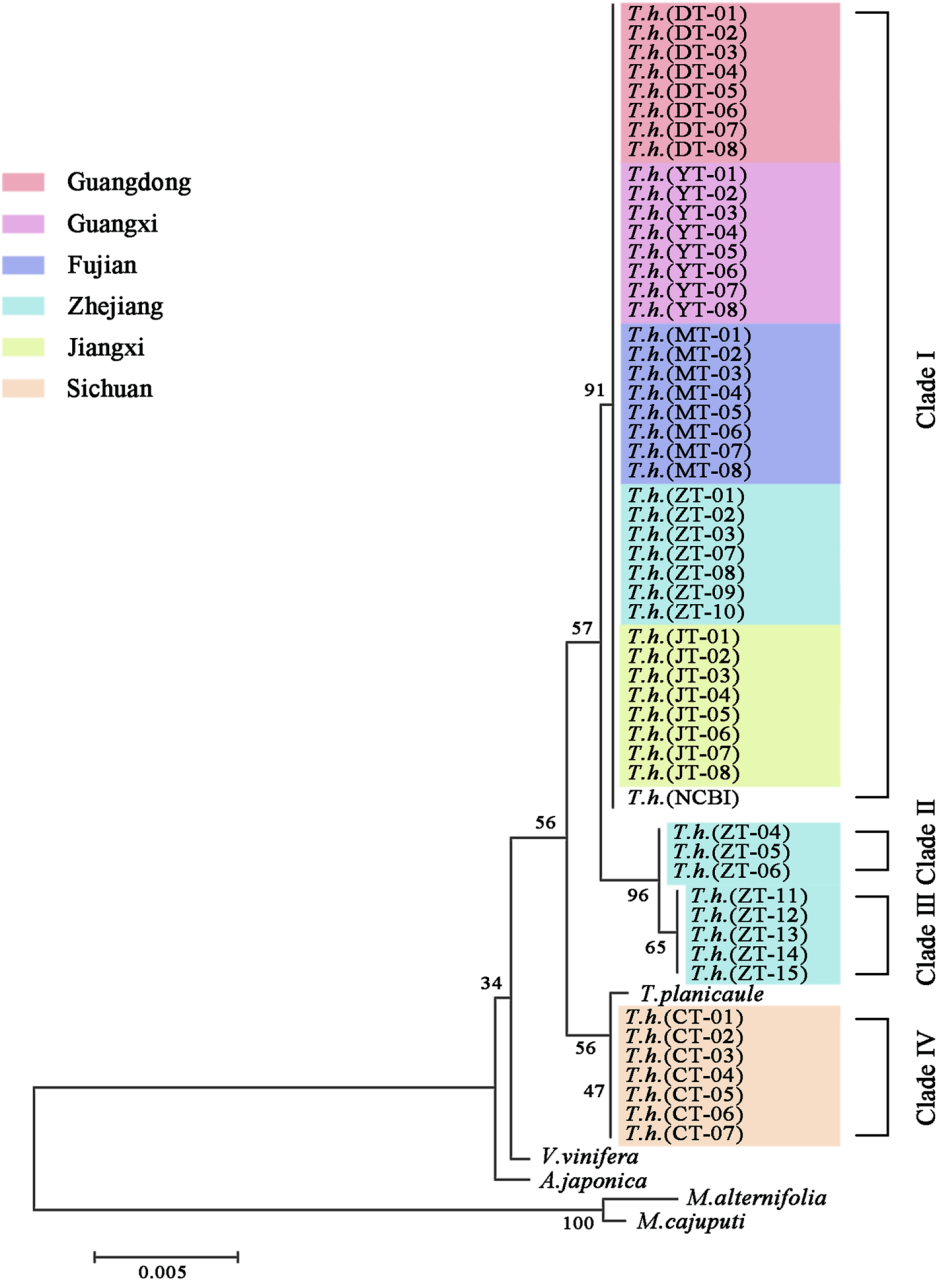
The Neighbor-Joining (NJ) tree analysis of 21 *T. hemsleyanum* samples from different regions and 3 representative Vitaceae species, based on the combination of *trnL* + *trnN* sequence. The six colors represent samples of *T. hemsleyanum* from six different provinces

This study also explored the genetic distance of intraspecific and interspecific variation within the *trnL* and *trnN* sequences of the *T. hemsleyanum* medicinal materials from different regions (Figs. 7B, C, D). The K2P distance of both *trnL* and *trnN* sequences among the 53 *T. hemsleyanum* samples ranged from 0.000 to 0.004, suggesting a significant barcoding gap among plants from different regions. For instance, the divergence value of *trnL* was highest (0.004) between the Jiangxi and Zhejiang regions and lowest (0.001) between the Jiangxi and Sichuan regions. The intraspecific genetic distance of *trnN* sequence between Jiangxi and the Sichuan region had a K2P value of 0.004, suggesting a barcoding gap. However, both of the two cp molecular markers failed to generate barcoding gap among species from Jiangxi, Fujian, Guangxi and Guandong province, indicating the inability of discriminating geographical origin from these regions by *trnL* and *trnN*. Moreover, the combination of *trnL* and *trnN* sequences revealed a lower intraspecific distance among different geographical origin of *T. hemsleyanum* than the single molecular marker (Fig. 7D). The intraspecific genetic distances based on *trnL*+ *trnN* sequences between Jiangxi and Zhejiang, Jiangxi and Sichuan, and among Jiangxi, Fujian, Guangxi, Guangdong and Zhejiang were 0.002, 0.003 and 0, respectively (Fig. 7D). The interspecific distance was greater among the Vitaceae species than that intraspecific distance of *T. hemsleyanum* samples, suggesting the developed DNA barcodes could be successfully applied for *T. hemsleyanum* species identification from other Vitaceae plants (Fig. 7D). The NJ tree analysis of the *trnL* and *trnN* barcodes revealed a clear distinction clearly among the different geographical origins of *T. hemsleyanum* plants (Figs. 7E, F). The *trnL*-based NJ tree generated four groups with different geographical origins, while *trnN*-based NJ tree only provided two clades of *T. hemsleyanum* plants. The Clade I of *trnL*-based NJ tree consisted of all *T. hemsleyanum* samples from Jiangxi, Fujian, Guangxi and Guangdong areas, seven samples from Zhejiang (ZT-01 ~ 03 and ZT-07 ~ 10) and one sample from Genbank (NCBI) with the bootstrap support value of 51. Clade II included *T. hemsleyanum* samples from Sichuan and *T. planicaule* samples. The rest of *T. hemsleyanum* samples from Zhejiang Province formed the Clade III (ZT-04 ~ 06) and Clade IV (ZT-11 ~ 15), respectively (Fig. 7E). The Clade II of the *trnN*-based NJ tree included all samples from Sichuan, while Clade I consisted of the samples from the other five regions and the sample (NCBI) from the Genbank. Although *trnL* barcode is more powerful in discriminating geographical origins of *T. hemsleyanum* than the *trnN* barcode, it failed to distinguish other Vitaceae species from *T. hemsleyanum* (Fig. 7E). Finally, we constructed the NJ tree based on the combination sequence of *trnL*+ *trnN* to determine the identification accuracy for *T. hemsleyanum* plants from different regions. Interestingly, this NJ tree divided the *T. hemsleyanum* plants into four groups and the dendrograms showed clear clustering pattern of geographically distribution. The six samples from Zhejiang province of ZT-04 to ZT-06，ZT-11 to ZT-15, and Sichuan samples of CT-01 to CT-03 each formed a separate group, while the other samples from Zhejiang province (ZT-01 ~ 03, ZT-07 ~ 10) and samples from other regions formed the fourth group (Fig. 8). Furthermore, the position of each Vitaceae species in the NJ tree based on a combination barcode was similar to phylogenetic trees based on the 70 protein-coding gene datasets (Figs. 5, 8). The NJ tree clearly showed that the *Tetrastigma* genus (*T. planicaule* and *T. hemsleyanum* samples) formed the main branch (bootstrap score, 56). They were significantly distinguished from the representative species of the Viteae tribe and Ampelopsideae tribe. This is an indication of the species identification potential of the combined molecular markers. These results demonstrate that the combined barcode strategy of *trnL*+ *trnN* derived from comparative chloroplast genomes is a potential molecular tool for the geographical origin discrimination of *T. hemsleyanum* in China.

## Discussion

### Chloroplast genome features of Tetrastigma and Vitaceae plants

The chloroplast genomes of the five *T. hemsleyanum* plants with different geographical origins as well as other representative species of *Tetrastigma* and Vitaceae exhibited a typical quadripartite structure containing a pair of IR regions, a single LSC region, and a single SSC region, which was similar to that of most vascular plants [38, 39]. The total length of the chloroplast genomes in this study ranged from 160,124 bp (*T. hemsleyanum* from Jiangxi) to 161,430 bp (*A. japonica*), that was consistent with the reported cp genomes from multiple other plants in Vitaceae, such as *A. grossedentata* (162,147 bp) and *V. davidii* (160,950 bp) [40, 41]. In spite of highly conserved cp genomes of angiosperms, gene loss and gain events continually occurred in certain species [42]. In our observations, the *ycf15* gene was only encoded uniquely in *T. hemsleyanum* and *A. japonica*, while it absented in *T. planicaule* and *V. vinifera* (Table 2). According to the previous studies regarding 10 Chinese wild Vitis species [43], the uniform loss of *ycf15* gene was indicated as one of the essential characteristics in Vitis plants. However, other reports from Ampelopsis species showed that the *ycf15* gene was disabled in *A. brevipedunculata* [44], and *A. humulifolia* [45]. These results further suggested relative complex regulatory functions of *ycf15* gene in the evolutionary of Vitaceae plants. Additionally, the *trnS-GCU* gene was merely encoded by *T. hemsleyanum*, while *trnG-GCC*, *trnG-UCC* and *trnV-GAU* were solely encoded by other three Vitaceae species, evidently reflecting the uniqueness of *T. hemsleyanum* in the family of Vitaceae. It is worth noting that the four special tRNA genes were all encoded by *Ampelopsis brevipedunculata* [44], resulting in a total of 31 distinct tRNA genes. The above results indicated that gene losses are not always dependable markers for phylogenetic relationships and further explorations focused on gene functions ought to be implemented and investigated urgently. With regards to the GC contents, chloroplasts from genus *Tetrastigma* (37.49–37.52%) showed slightly higher ratio than that of most *Ampelopsis* species (37.33–37.37%) [44, 45] and *Vitis* species (37.05–37.40%), except that of *V. romanetii* (38.28%) exhibiting an unusually large genome in size (232,020 bp) [43]. It is common knowledge that GC base pair is more stable than the AT. Accordingly, the increase of GC content in *Tetrastigma* species could potentially improve the stability of chloroplasts, consequently contributing to their adaptation to some harsh growing environments such as rocks crevices.

The expansion and contraction of the IR boundary is one of the main driving forces of changes in chloroplast genome size [46]. Except for the *rpl2* gene, there was no significant variation among the *T. hemsleyanum* chloroplasts from different regions. In addition, only slight IR expansions and contractions were found in every border of *T. hemsleyanum* chloroplasts, further confirming their conserved traits of IR boundary (Fig. 3). However, the analyzed results indicated the location and the intron number of *rpl2* gene were diverse in *T. hemsleyanum* cp genomes from different regions. The *rpl2* gene of *T. hemsleyanum* cp genome from Sichuan, Fujian and Jiangxi *T. hemsleyanum* cp genomes showed one intron and located in IRA completely, which was consistent with the reports of previous researches on *T. planicaule* [47] as well as other Vitaceae species [45, 48]. Remarkably, *T. hemsleyanum* chloroplasts from Guangxi and Zhejiang provinces possessed two introns that located across the border of JLA, which was not common in Vitaceae plants (Fig. 3). Nevertheless, several lineages of dicotyledons, including Saxifragaceae and Convolvulaceae, have even been reported to lose the intron of *rpl2* gene independently [49], which was regarded as the main characteristic of core members of Caryophyllales [50]. Moreover, shrinkage and expansion of the IR boundary could also trigger the duplication of genes or the generation of pseudogenes in angiosperms chloroplast genome [51]. Among the analyzed Vitaceae species in this study, only the cp genome of *V. vinifera* possessed a pseudogene *ycf1* located in IRB region completely. The pseudogenizations of *ycf1* gene were also documented in other Vitis species [43]. However, the similar event was not observed in Ampelopsis and *Tetrastigma* plants. Previous studies have suggested that *ycf1*, a well-known gene with the most variable sites in cp genome, could be a promising DNA barcode with better performance than these current universal barcodes [52]. Nevertheless, our results showed that *ycf1* region was not the marked hotspot with the most variation sites during the cp genome comparison analysis among *T. hemsleyanum* plants and other four Vitaceae species. Therefore, the function of *ycf1* gene and its specific role in phylogenetic relationship of Vitaceae need to be further elucidated with more cp genomes.

The adaptive evolution of cp genome genes represented valuable tool for exploring the variation of gene function, structural change and evolutionary of species [53]. The pairwise Ka/Ks values have been extensively used as an efficacious indicator to reveal positive selection pressure and adaptive evolution rate of species [54]. For the protein-coding genes with RNA editing sites in *T. hemsleyanum* chloroplasts in our study, the majority (91.67%) of Ka/Ks values exhibited a range from 0 to 0.5, which was in accordance with the previous researches on some Vitaceae plants [44]. The lowest Ka/Ks value (0) was observed within genes encoding subunits of photosystem II (*psbF* and *psbL*) and protease (*clpP*). While the most salient Ka/Ks values happens on *matK* and *rps16*, which encoded maturase and small subunit of ribosome. The higher Ka/Ks values of *matK* and *rps16* indicated that these two genes might be more sensitive to the environment changes. The combination approach of *matK* and *rps16* sequences has been successfully applied to distinguish the primitive species of *Polygonatum* species [55]. In addition, the *matK* gene was also considered to be necessary for the survival of plant cells, and its expression required seriously strict regulation to prevent pernicious effects [56]. The previous data have suggested that the evolution of *matK* region was considered as the fastest gene in chloroplast genome, providing a powerful marker for genetic classification of jewel orchid accessions in Vietnam [57]. Herein, the *matK* gene with high Ka/Ks value could also be conjectured to be potential for species identification and genetic relationship study among Vitaceae plants.

### Phylogenetic analysis and taxonomic implications within family Vitaceae

The grape family Vitaceae is an economically important family of angiosperms containing 16 genera and about 950 species. The Phylogenetic analysis based on the chloroplast genome sequences strongly supported the position of the Vitaceae as the earliest diverging lineage of rosids [58]. In addition, the Vitaceae family could be divided into five tribes by both nuclear and chloroplast genomic data, including Ampelopsideae, Cayratieae, Cisseae, Parthenocisseae, and Viteae, respectively [59]. Our phylogenomic analysis recovered three well supported lineages within Vitaceae (Fig. 8) that correspond to the three tribes reported in previous study [37]. The evolutionary relationships constructed in our study were consistent with those of previous researches [37, 60, 61], further confirming the advances of whole chloroplast genome analysis on the phylogenetic resolution in certain lineages. The molecular phylogenetic analysis of Vitaceae was initially investigated utilizing several plastid genes, including the *trnL-F*, *atpB-rbcL*, *trnC-petN*, *trnH-psbA*, spacer and *rps16* intron [61, 62]. A phylogenetic tree of Vitaceae with 417 single-copy nuclear genes was reconstructed from transcriptomes of 15 Vitaceae species, providing robust support for the deep relationships of the grape family and indicating the phylogenetic utility of transcriptome data for plants over a time scale [63]. Subsequently, the deep relationships of Vitaceae family was explored by skimming the chloroplast and mitochondrial genomes for 27 taxa, supporting the division of five major clades of the family: the Vitis-Ampelocissus clade, the Parthenocissus-Yua clade, the core Cissus clade, the Cayratia-Cyphostemma-Tetrastigma (CCT) clade, and the Ampelopsis-Rhoicissus clade [37, 64]. It is interesting to note that the plants of tribe Cayratieae, including genera *Causonis*, *Cayratia*, *Cyphostemma*, *Pseudocayratia* and *Tetrastigma*, exhibited larger genomes than other four tribes in grape family [65]. Specially, five chloroplast molecular markers were employed to the phylogeny and biogeography of Cayratia, confirming the monophyly of the CCT clade and further suggesting its geographical origin of continental Africa in the late Cretaceous [60]. Furthermore, phylogenetic relationships within 72 *Tetrastigma* species recognized six strongly supported clades on the basis of ten plastid DNA regions, which do not completely correspond to their geographical distributions [66]. Nevertheless, the genus of Cayratia has been proved to be divided into three branches, relating to their geographical distribution [67]. The genus of *Cayratia* in Africa formed an independent group, while the other two groups were consisted of samples from Asia and Australia, which reflected the great impacts of different geographical origins on phylogenetic classification [68]. In this study, we determined the complete cp genomes of *T. hemsleyanum* from five provinces and constructed a comparative analysis with representing plants from tribe Cayratieae, which enriched the genome database of *T. hemsleyanum* and provided the basic data for improving the phylogenetic relationships among *Tetrastigma* species with better resolution. However, the sample sources in our research were all from China in Asia. Studies including a wider sampling area should be carried out across the genus to further elucidate the deeper relationships within *Tetrastigma* and Cayratieae.

### Development of molecular markers for efficient species classification in Vitaceae

The complete chloroplast genomes have proven to be powerful tool to resolve evolutionary relationships among plant species, and provide valuable information for molecular species authentication [69]. Compared to the potential markers from nuclear genome, the chloroplast genome derived markers harbored rich gene copies in plants and sufficient inter-specific divergence coupled with low intra-specific variations [70]. The identified highly variable regions of cp genomes could be developed as efficient DNA barcodes and used extensively in rapid species identification and large-scale phylogenetic analysis [71, 72]. The previous reports have confirmed that seven DNA barcodes selected based on the basis of the cp genomes of *Pterocarpus* species displayed better discernibility when comparing with the universal barcodes of *rbcL*, *matK*, *trnH-psbA* and *trnL-F* [73, 74]. In our study, a total of five hypervariable regions across four complete cp genomes of Vitaceae species were identified, of which four were intergenic regions (*rps16-trnQ*, *psbM-trnD*, *psbZ-trnfM* and *ycf3-trnS*) and one was protein coding gene (*ycf1*) (Fig. 6A). In addition, the IR regions of Vitaceae plants exhibited significant lower sequence divergence than the SSC and LSC regions, which was consistent with the results from other plants [75]. It is a very common phenomenon in the cp genomes of most angiosperms, where the intergenic spacers contained more sequence variants than the coding genes. The developed molecular markers with higher inter-specific levels have been widely reported in other studies and exhibited excellent discriminating ability in investigating taxonomy and molecular phylogeny, such as Pulsatilla of Ranunculaceae [76] and Rhodiola of Crassulaceae [77]. It is worth noting that the three intergenic regions, *rps16-trmQ* [78], *psbM-trnD* [40] and *psbZ-trnfM* [79] found in our results were also reported to be candidate DNA barcodes for plant identification and phylogenetic relationships analysis in Echinacanthus, Myrtales and Zygophyllum species, respectively. The deep phylogenetic relationship within the tribe Cayratieae and family Vitaceae still remains uncertain due to high morphological similarity and less molecular data. Furthermore, the dried root from *T. hemsleyanum* bears a striking morphological resemblance to that from closely related species of Cayratieae and Vitaceae, which caused the emergence of adulterant and counterfeit drugs in the market and led to potential hazard to health and clinical efficiency. The molecular phylogeny based on the combination of three chloroplast markers suggested the split of genus *Vitis* into three clades and supported a relatively recent and intense gene flow in species from different regions [80]. The universal DNA barcode *ITS2* was reported to distinguish *T. hemsleyanum* from its adulterants, providing an effective and accurate identification strategy for this endangered herb [12]. However, we failed to sequence and amplify the *ITS2* gene with the DNA template extracted from the processed roots of *T. hemsleyanum*, indicating the potential destruction on nuclear genomes during the processing of crude drugs (data not shown). The destroyed nuclear DNA brought the limitations of universal nuclear DNA barcode sequences for distinguishing processed medicinal plants. Contrast with the nuclear barcode *ITS2*, the developed chloroplast genome markers of *trnL* and *trnN* could be successfully amplified with the genomic DNA from the dried root of *T. hemsleyanum*, suggesting the chloroplast genome was more stable than the nuclear DNA during the processing of medicinal plant. Therefore, the identified mutational hotspots regions and according primer set were believed to help to distinguish the taxa in the genus level, which would provide a credible approach to identify related species and assess the interspecific phylogenetic relationships among Vitaceae plants.

The hypervariable region worthy of special attention was *ycf1*, which exhibited the highest Pi value among the coding genes in this study (Fig. 6A). The DNA barcode of *ycf1* has been confirmed the excellent ability in identification of *Fritillaria* species [81] and phylogeny reconstruction of Primula species [82]. The comparative analysis of the *ycf1* gene in four Vitaceae plants revealed that fewer SNP sites and Gaps were shown between *T. hemsleyanum* and *T. planicaule* while more variations were presented between *A. japonica* and *V. vinifera* (Table 6). These results indicated that the *ycf1* was more reliable for the research on phylogenetic relationships in Vitaceae plants than the species identification of related plants within *Tetrastigma*. In addition, the PCR amplified length of the currently determined three hypervariable regions of *psbM-trnD*, *psbZ-trnfM* and *ycf1* were less than 1000 bp, which could result in the high success rates of amplification and sequencing. Therefore, these three diversity regions presented candidate barcoding sequence, which might be helpful to plant identification, systematic investigation of Vitaceae and evaluating the phylogenetic relationships among the tribe of Cayratieae.

### Geographical origin discrimination strategy for medicinal plants

The quality of medicinal plants depends on various factors with significant contributions of genetic impacts and geographic location. The development of geo-authentic Chinese medicinal material was closely associated with cultivated outstanding genetic mutants and eligible local environmental impacts, leading to the production of famous crud drugs with higher quality and price in the market [83]. With the increasing global demand for plant medicines, great numbers of medicinal species have been cultivated in different areas to generate multiple genetic populations with similar morphological features [84]. Due to the significant advantages of genuine medicinal material from geo-authentic producing areas, it was more likely to be adulterated with lower-price counterparts from other different regions [26]. However, the geographical origin of herbal medicines has been indicated as a crucial factor influencing the quality and potential treatment efficacy of the medicinal materials, which could be attributed to the variations in the environmental conditions and genetic reasons [85]. Zhang et al. [86] confirmed that there were obvious differences in chemical components of dandelions from four different geographical regions by metabolomics analysis, especially in phenolic compounds. To ensure the health benefits and clinical effectiveness of herbal medicine to consumers, it is necessary to develop strategies for the recognition of geographic origin crud drugs. Since the obvious differences on the content of chemical constituent and plant genetic populations, several analytical approaches of DNA and chromatographic analysis have been extensively applied to determine the geographical origin of medicinal plants and foods [87]. The HPLC similarity analysis and content of alkaloids was indicated as valuable tools for differentiating the geographical origin of the Fuzi samples [88]. The excitation-emission matrix fluorescence and chemometric strategies have also been considered as promising methods for distinguishing the geographical origin of *Gastrodia elata* [89]. Compared with the chemotaxonomical investigations, the DNA analysis based on either unique sequence regions or DNA polymorphism from genetic markers represented alternative approaches to identify plant populations and authenticate plant species. The amplification of unique gene fragment exhibited a rapid and easy method to identify the geographical authenticity of *Scrophularia ningpoensis* [67]. The SSR markers were indicated as suitable tool for assessing genetic diversity and population structure of spinach germplasm, which clearly separated the accessions with different geographical origins [90]. Recently, the combination of DNA molecular markers and chemical analytical techniques has been successfully used to distinguish the geographical origins of traditional Chinese medicines. The integration of microsatellite markers and chemical analysis could discriminate the *Moutan Cortex* from different sources and geographical origins [91]. The ISSR fingerprinting combined with FTIR spectrum analysis established a rapid and efficient approach to determine the *Cassia tora* populations with different eco-geographical origins [92]. The development of efficient geographical tracing system of medicinal plants would significantly contribute to the protection of genuine plant genetic resources and improvement for quality control of herbal drugs.

Since the significantly differences on the crude drug quality and clinical efficiency of *T. hemsleyanum* from different regions in China, a variety of identification approaches have been reported for discriminating geographical origins of *T. hemsleyanum*, including determining strategies based on macroscopic analysis, spectroscopic technology, chromatographic fingerprint and bio-activity evaluation. Previous study has reported that the root tuber of *T. hemsleyanum* from Zhejiang and Guangxi province could be successfully distinguished by analyzing the external characteristics of tuber and the typical micro-structures of powder, thus providing an intuitive and simple approach for plant origin determination [93]. However, pharmacognosy-based identification cannot accurately identify the processed decoction pieces of *T. hemsleyanum* from the above two aspects. Li et al. [94] effectively distinguished the *T. hemsleyanum* samples from Zhejiang, Yunnan and Guizhou Province using a combined identification approach based on HPLC fingerprints and the random forest (RF) algorithm analysis. Machine learning algorithm has been widely used for spectral data processing to discriminate medicinal herbs from different habitats. A dual-mode microscopic hyperspectral imager (DMHI) system has been developed using the combined dataset of RMHI and FMHI modes for hyperspectral detection of the origins and varieties of *T. hemsleyanum*, obtaining a prediction accuracy as high as 97.5% of both origins and varieties [95]. Besides, the near-infrared spectroscopy (NIRS) combined with deep learning models also exhibited potential capability to distinguish the medicinal plant *T. hemsleyanum* among different origins [96]. However, the processing of crude drugs and growth years and harvesting seasons of medicinal materials can significantly affect the geographical origin identification methods based on chemical contents and compositions, thus generating inaccurate results. Furthermore, deep learning is complex, time-consuming, and associated with a low signal-to-noise ratio (SNR), instability, and spectrum peaks overlap, thus limiting its application. In contrast, the origin discrimination approaches based on DNA markers could target the direct carrier of genetic information in plant populations, thus providing an alternative strategy with higher stability than those based on chemical analysis. Therefore, this study provides an efficient method for distinguishing the geographical origins of *T. hemsleyanum* based on DNA barcodes from the cp genome from different regions. The developed specific DNA barcodes and their combination divided the 53 *T. hemsleyanum* samples from six provinces into four haplotypes and successfully classified a sample from Genbank into Clade I. The specie of *T. hemsleyanum* exhibited genetic patterns characteristic of long-term separation in multiple refugia and lower levels of interpopulation gene flow, indicating that the genetic population divergence largely driven by mutation or drift, further supporting the genetic stability of *T. hemsleyanum* samples in this study [1]. Previous study on lineage diversification reported that *T. hemsleyanum* was consisted of two major cpDNA lineages, Southwest (SW) and Central-South-East (CSE) China, consistent with our grouping results [97]. Herein, the *T. hemsleyanum* samples from CSE China were gathered into a gigantic branch, while those from Sichuan Province in SW China were grouped in another cluster. Furthermore, Besides the DNA molecular barcoding strategy, other molecular markers of RAPD and SRAP also displayed the potential capacity in determining the geographical origins of *T. hemsleyanum*. For instance, Yin et al. [98] have sifted out 10 pairs of RAPD primers for PCR amplification of 64 samples of *T. hemsleyanum* from 14 provinces in China. They revealed abundant genetic diversities of *T. hemsleyanum* germplasm resources and significant complexity of geographical distribution. However, the RAPD cluster analysis was inconsistent with the geographical distance of the provenance, requiring further revisions for primers for geographical origin determination. Notably, SRAP markers divided the same 64 *T. hemsleyanum* samples into nine groups, which exhibited certain different results with that from RAPD analysis [99]. Consortium for the Barcode of Life [100] has confirmed that combining barcodes has a better identification efficiency than a single barcode. Herein, the combination of *trnL* + *trnN* barcode divided the *T. hemsleyanum* plants into four groups, thus effectively identifying the genetic populations of *T. hemsleyanum* samples from Zhejiang and Sichuan provinces. It is worth noting that the samples of *T. hemsleyanum* in Zhejiang Province exhibited three haplotypes. One of them was consistent with the samples from Fujian, Guangdong, Guangxi and Jiangxi provinces, and the other two haplotypes were unique to Zhejiang Province. Besides, all samples from Sichuan Province were clustered into a particular branch. However, our results failed to distinguish the strains in each of other provinces concretely, generating a clade with *T. hemsleyanum* samples from Zhejiang, Jiangxi, Fujian, Guangxi, Guangdong and Genbank database. A promising approach for the accurate traceability of *T. hemsleyanum* from different regions should be established urgently.

Multiple universal barcode markers have been proposed for recognizing species at a genera and family level, such as matK, *rbcL* [100], *ITS2* [101], *trnH-psbA* [102] and *trnL-F* [103] with the length of DNA sequences between 400 and 1000 bp. Similarly, it is also applicable to trace various geographical origins of species with universal barcodes. The method based on universal DNA barcode *ITS2* suggested that *T. hemsleyanum* from Zhejiang province had a unique genetic status, exhibiting potentiality for the plant population and geographical origin discriminating between Zhejiang and other provinces [12]. Nevertheless, there is no doubt that low discriminatory power is inevitable for universal DNA barcodes, especially in tracing intraspecific geographical origins. Increasing number of case studies have indicated that the universal DNA barcodes have lower divergence and poor discriminatory power [12]. The mutation events in the chloroplast genome are not universally randomly distributed within the sequence and are concentrated in certain regions forming the “hotspot” regions [104]. Comparison of the chloroplast genome sequences is an effective strategy to identify the mutation hotspots and these highly variable regions can be used as the specific DNA barcodes. This view could be explained by the fact that universal barcodes possessed lower nucleotide diversity (Pi) values (0.00075–0.0025) while the higher Pi values (0.003) presented in specific barcodes of *trnL-CAA* and *trnN-GUU* (Supplementary Fig. 3). This is also the reason that we performed the work to identify more informative DNA regions for geographic identification of *Tetrastigma hemsleyanum*.

SSRs have become a new molecular marker technology and extensively applicated in plants genetic diversity, gene mapping and variety identification with the characteristics of strong polymorphism, co-dominance, high universality and good stability [105]. The distribution of chloroplast SSR characteristics exhibited taxon specificity in Cyatheaceae species, which provided valuable phylogenic information at the genus level [106]. Furthermore, the SSR primers designed based on different *Gracilaria tenuistipitata* chloroplast genomes from various regions could separate the samples into two main geographical regions, which significantly contributed to the mass cultivation of seaweeds with high economic potential [107]. The developed program, ChloroSSRdb focused on the application of chloroplastic SSRs from Viridiplantae, thus providing useful resources in developing genetic markers and phylogenetic analysis [108]. The ISSR molecular markers divided the germplasm resources of *T. hemsleyanum* from the main distribution areas of China into 4 Clades, among which Zhejiang samples were all clustered in the Clade I [109]. Another report showed that ISSR and SRAP markers could cluster the wild accessions of *T. hemsleyanum* into four groups (similarity coefficient level, 0.75) [14]. All the wild populations from Zhejiang were highly distinct for ISSR polymorphism and formed a separate cluster, while those from the other three clusters consisted of *T. hemsleyanum* samples from Guangxi, Jiangxi and Hunan. This study also found abundant SSRs in the cp genomes of *T. hemsleyanum* plants. Furthermore, significant differences were identified on SSR numbers and types in the *T. hemsleyanum* cp genomes among five samples from different regions, mainly distributed in quantities of mononucleotide repeats and types of repeat unit (Fig. 4A). Meanwhile, a comparison among four Vitaceae species including *T. hemsleyanum* from Jiangxi has revealed great discrimination in SSR counts and types between different tribes, such as tribe Cayratieae, Ampelopsideae and Viteae, suggesting the potentiality of SSRs in species discrimination and classification (Fig. 4B). However, most of current studies focused on the interindividual genetic variation of *T. hemsleyanum*, thus limiting the use of SSR markers in determining the genetic populations from different regions. Therefore, the SSRs fingerprints should be systematically constructed for geographical origin identification of *T. hemsleyanum* based on the present comparative analysis of cp genomes from different regions.

## Materials and methods

### Plant material and DNA extraction

The plant materials of *T. hemsleyanum* were collected from six different provinces in China (Supplementary Table 1) and identified by Dr. Yuqing Ge of Zhejiang Chinese Medical University. The plant specimens were deposited at Medicinal Herbarium Center of Zhejiang Chinese Medical University (https://yxy.zcmu.edu.cn, Herbarium Code: MHCZCMU, collector: Rubin Cheng, biothcheng@hotmail.com). The detailed voucher numbers of *T. hemsleyanum* with different geographical origins were listed in Table S1. Total genomic DNA was extracted from the fresh and healthy leaves of T. hemsleyanum using a modified cetyltrimethylammonium brofmide (CTAB) method [110]. The final DNA integrity and concentration were assessed by electrophoresis on 1.0% agarose gel and Nanodrop 2000 Spectrophotometer (Thermo Fisher Scientific, United States).

### Sequencing, genome assembly and annotation

The paired-end (150 bp) sequencing of the DNA libraries was conducted on the Illumina HiSeq 2500 platform, generating about 2.4 GB of raw data for each sample. Then the quality of paired-end Illumina reads was assessed with FastQC, and the low-quality reads were removed using Fastp. The filtered reads were assembled de novo using metaSPAdes with the complete cp genome of *T. hemsleyanum* (NC_029339) as reference and the protein-coding genes, mRNA genes, tRNA genes were annotated by GeSeq annotation tool [111]. The CPGAVAS2 software also used to annotate protein-coding genes [112]. BLAST was further used to correct the annotation of chloroplast genome manually. The circular chloroplast genome map of *T. hemsleyanum* collected from Jiangxi Province were drawn by OrganellarGenomeDRAW (OGDRAW) tool [113]. Finally, the fully annotated cp genomes were deposited at the GenBank database (Supplementary Table 1).

### Comparative analysis of chloroplast genomes and identification of hypervariable regions

MEGA 7.0 [114] was used to analyze the genome feature and Codon W software was used to investigate the distribution of codon usage using the RSCU value [115]. The IR/SC boundary locations in five samples of *T. hemsleyanum* and three representative Vitaceae species were compared using IR scope [116]. As for the repeats analysis, long repeats of four different type (forward (F), palindromic (P), reverse (R), and complementary (C)) were identified by REPuter, with hamming distance 3, minimal repeats 30 and maximum computed repeats 50 [117]. Simple sequence repeats in Vitaceae species were detected by MISA, setting parameters as 10 for mononucleotide SSRs, 5 for dinucleotide SSRs, 4 for trinucleotide SSRs, 3 each for tetranucleotide, pentanucleotide and hexanucleotide SSRs [118]. To predict the number of RNA editing sites, the PREP-Cp program was employed with a cutoff value 0.8 [119]. For the identification of hypervariable regions within five samples of *T. hemsleyanum* and among four representative species of Vitaceae, we aligned cp genome sequences using MAFFT [120] and evaluated the sequence divergence among Vitaceae species through a sliding window analysis in DnaSP v6 [121]. The parameters of sliding window analysis were set as window length for 800 sites and the step size of 200 sites.

### Ka/Ks and positive selection analyses

In order to analyzed the Ka and Ks substitution rates and Ka/Ks ratio, *Melaleuca cajuputi* was compared with *T. hemsleyanum* (Jiangxi Province) in 24 protein coding genes. The alignment was carried out by MAFFT v7.037b [120], and the calculation of the value of Ka/Ks was implemented by DnaSP v6 [121].

### Primier design and PCR amplification

Based on conserved nucleotide sequences at both ends of mutation hotspots, 5 pairs of specific primers were designed by Primer Premier 5 (Supplementary Table 2). PCR was performed in 50-μL reactions consisting of 3 μL of genomic DNA, 5 μL of dNTP Mix and 10× LA PCR Buffer, 2.5 μL of forward and reverse primers (25 μmol/L), 0.5 μL of LA Taq (Takara) and ddH2O supplemented to 50 μL. PCR amplification was carried out by Veriti Thermal Cycler (Applied Biosystems) with the following program: 5 minutes at 94 °C for initial denaturation; denaturation 94 °C, 45 seconds; 33 cycles consisting, annealing temperature 53 °C - 58 °C, 30 seconds, extension temperature 72 °C, 45 seconds, final 10 minutes extension at 72 °C, 4 °C low temperature save. PCR products were examined by electrophoresis on 1.0% Agarose Gel and visualized with 4S GelRed Nucleic Acid Stain (Sangon Biotech, China). Finally, optimum annealing temperature of *trnL* and *trnN* sequences was determined as 56 °C. The synthesis of primers and Sanger sequencing were conducted by Sangon Biotechnology and Shanghai Sunny Biotechnology Co., Ltd., respectively.

### Multiple sequence alignment and data analysis

The sequencing results were performed using BioEdit 7.0 [122] and aligned by MAFFT 7.0 [120] to quantify the sequence length and base composition. To count the variant information, the aligned sequences were analyzed by MEGA 7 [114]. Based on the single and combination sequences, the intraspecific genetic distance of *T. hemsleyanum* between Jiangxi Province and other regions, as well as the interspecific genetic distance among representative species of Vitaceae were calculated using MEGA 7 with Kimura 2-parameter (K2P) distance model.

### Phylogenetic analysis and effectiveness of marker discriminatory

The maximum likelihood (ML) tree and maximum parsimony (MP) tree were constructed using 70 conservative protein-coding genes of five *T. hemsleyanum* complete cp genomes sequenced in this study and 10 additional publicly available sequences that we downloaded from the NCBI. Among these 15 species, *Melaleuca alternifolia* and *Melaleuca cajuputi* were chosen as outgroups. The ML tree was constructed based on the K2P model with 500 bootstrap replications. Similarly, MP tree was obtained from MEGA 7 [114] under default parameters with 500 bootstrap replications. Based on the K2P distance model, the single and combination *trnL* or *trnN* sequences of Vitaceae species were used to construct the NJ trees with 1000 bootstrap replications.

## Conclusions

This study provides 5 complete chloroplast genome sequences of *Tetrastigma hemsleyanum* with different geographical origins in China, and presents a comparative analysis of cp genomes with other representing species from family Vitaceae. The chloroplast genome structure of *T. hemsleyanum* samples from different regions and other Vitaceae plants was highly conserved. However, IR expansion and contraction was observed among cp genomes of *T. hemsleyanum* from different areas, resulting in cp genomes of different sizes. In addition, significant differences in SSR types and numbers were identified in the cp genomes of different *T. hemsleyanum* samples as well as Vitaceae species, providing valuable genetic information for the development of species identification strategy and geographical origin determining system. Phylogenetic analysis revealed the five *T. hemsleyanum* plants clustered together to form a stable monophyletic group, exhibiting sister relationship with *T. planicaule* to compose the tribe of Cayratieae. A total of five highly variable regions with significant differences between *T. hemsleyanum* and other Vitaceae species were identified and may be applied as potential markers for species identification and further phylogenetic relationship analysis in family of Vitaceae. Furthermore, the cp molecular markers of *trnL* and *trnN* were successfully built based on the hotspots among *T. hemsleyanum* cp genomes with different regions. The combination of *trnL* and *trnN* could divide the *T. hemsleyanum* plants from six different provinces into four genetic groups, three of which were found in Zhejiang Province. These results obtained in this study would contribute to the understanding of phylogenetic relationship and systematic evolution of Vitaceae plants, and provide valuable molecular approaches to discriminate the geographical origins of *T. hemsleyanum* and protect the diversity of *T. hemsleyanum* germplasm resources.

## Supplementary Information

**Additional file 1.**

**Additional file 2.**

## Data Availability

The datasets generated for this study can be found in National Center for Biotechnology Information (NCBI) under the accession numbers: MW375707-MW375711; MZ995433-MZ995472; OK058531-OK058532; ON561826-ON561890; ON624114.

## Abbreviations

BLAST: Basic local alignment search tool
cp: Chloroplast
DNA: Deoxyribonucleic acid
IR: Inverted repeat
LSC: Large single copy region
SSC: Small single copy region
SSR: Simple sequence repeats
