# Supplementary material for: The complete chloroplast genomes of Tetrastigma hemsleyanum (Vitaceae) from different regions of China: molecular structure, comparative analysis and development of DNA barcodes for its geographical origin discrimination
Source: BMC Genomics. 2022 Aug 26;23:620. doi: 10.1186/s12864-022-08755-7 (PMC9412808; doi:10.1186/s12864-022-08755-7)
Supplement: Supplementary file 1 — Additional file 1. [file 12864_2022_8755_MOESM1_ESM.docx]

Supplementary Material Figures


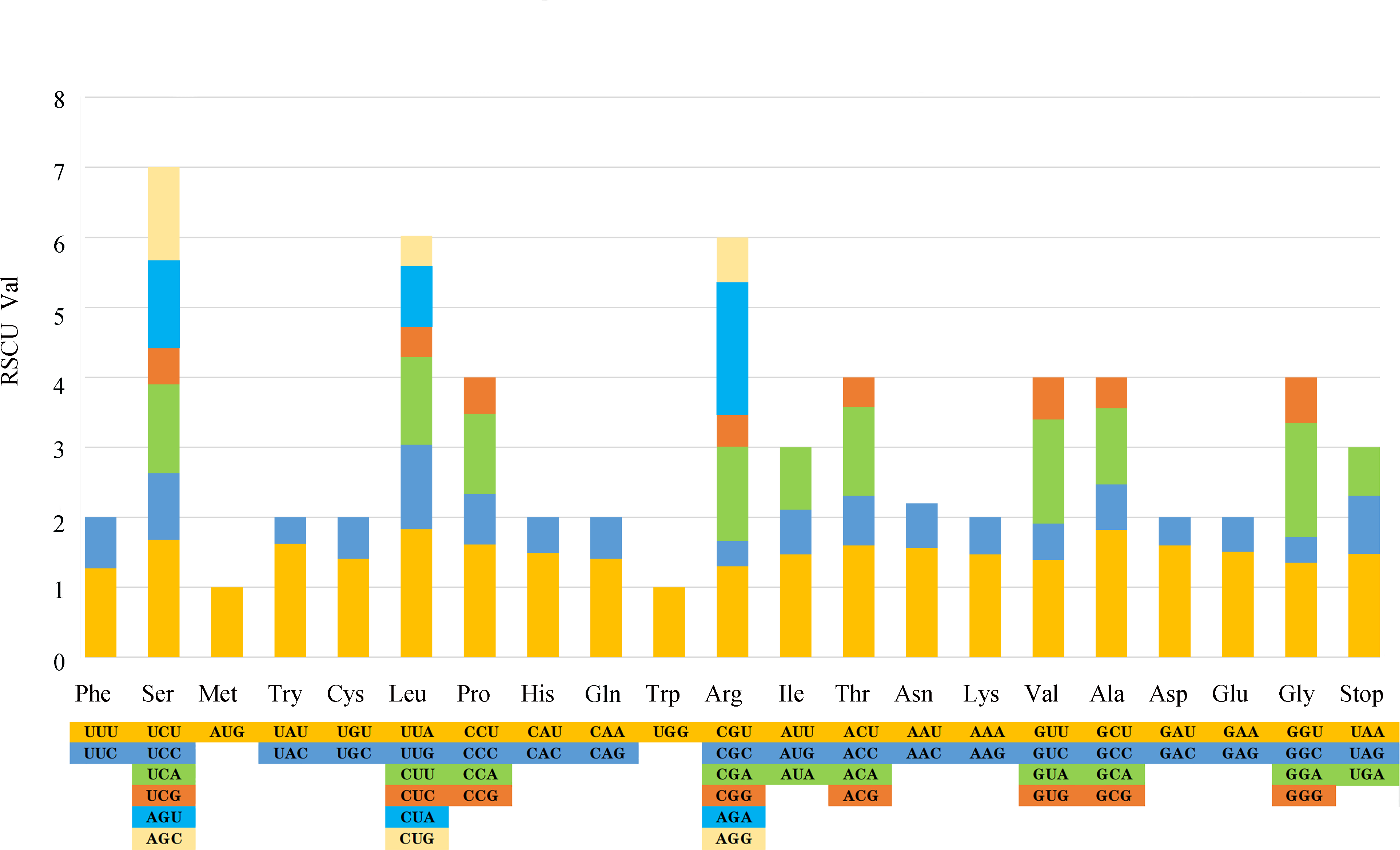


**Supplementary Figure 1.** The RSCU value of the 20 amino acids and stop codons in all the protein- coding genes of the chloroplast genome of *Tetrastigma hemsleyanum* sample from Jiangxi Province.

Supplementary Material


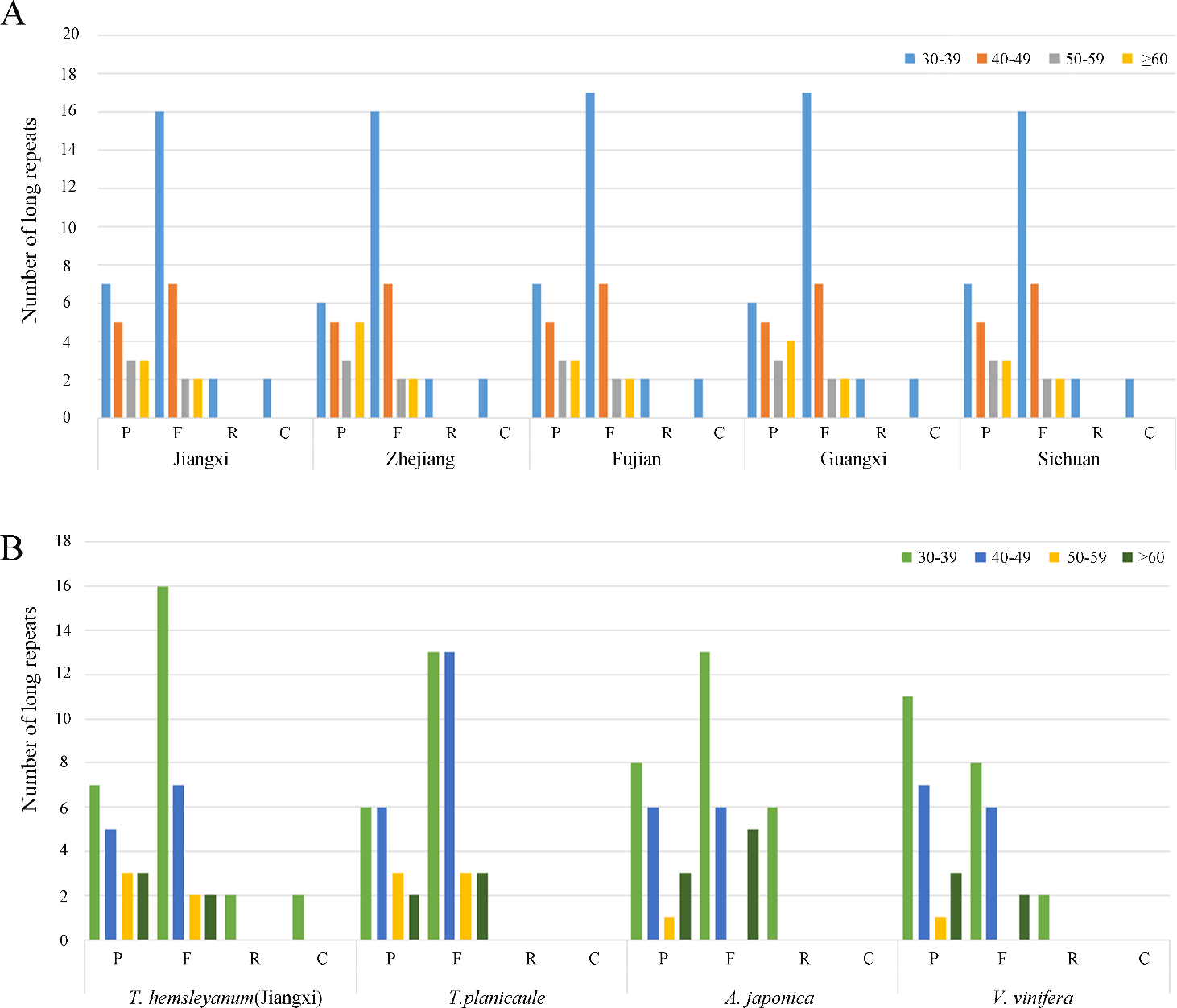


**Supplementary Figure 2.** Analysis of the long repeats in cp genomes of Vitaceae plants and *Tetrastigma hemsleyanum* species with different geographical origins. **(A)** The number, length and type of the long repeats identified in five samples of *Tetrastigma hemsleyanum*. (**B**) The number, length and type of the long repeats detected in *Tetrastigma hemsleyanum* (Jiangxi Province), *Tetrastigma planicaule*, *Ampelopsis japonica* and *Vitis vinifera* cp genomes.

Supplementary Material


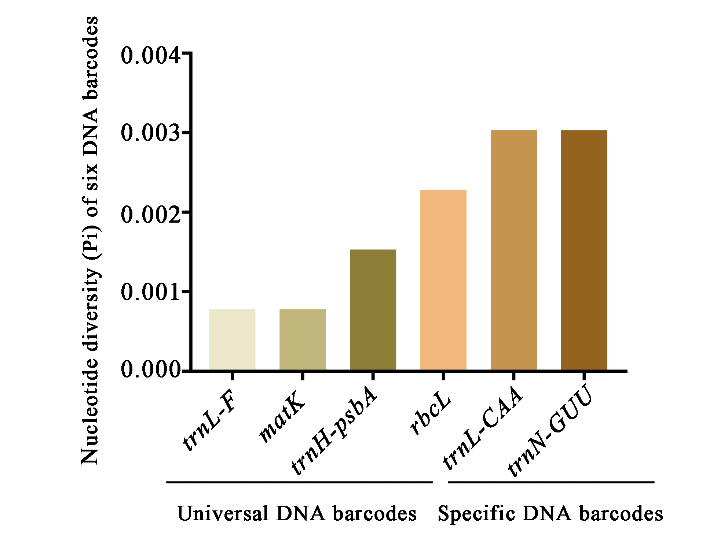


**Supplementary Figure 3.** Comparison of nucleotide diversity (Pi) between universal DNA barcodes and specific DNA barcodes in *Tetrastigma hemsleyanum* cp genomes.

Supplementary Material


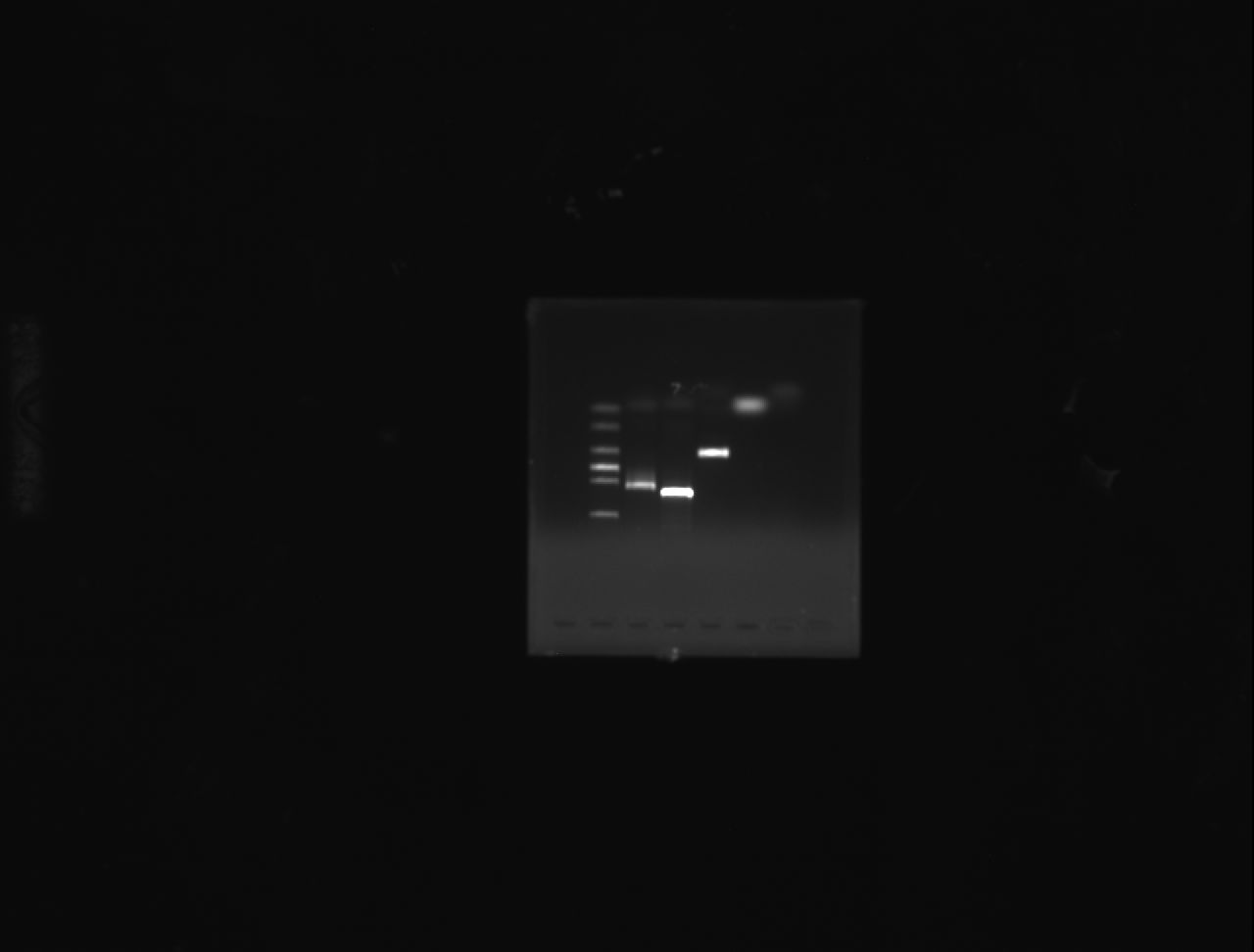


**Supplementary Figure 4.** Original agarose gel electrophoresis of PCR products of five developed DNA barcodes from *Tetrastigma hemsleyanum* of Zhejiang Province.
