# Supplementary material for: The complete chloroplast genomes of Tetrastigma hemsleyanum (Vitaceae) from different regions of China: molecular structure, comparative analysis and development of DNA barcodes for its geographical origin discrimination
Source: BMC Genomics. 2022 Aug 26;23:620. doi: 10.1186/s12864-022-08755-7 (PMC9412808; doi:10.1186/s12864-022-08755-7)
Supplement: Supplementary file 2 — Additional file 2. [file 12864_2022_8755_MOESM2_ESM.docx]

Supplementary Material Tables

Supplementary Table 1. The information of sample collection and DNA sequencing of T. hemsleyanum in five regions.

| **Sample No.** | **Location** | **Latitude (N)** | **Longitude (E)** | **Raw reads** | **Raw Bases** | **Genbank accession number** |
| --- | --- | --- | --- | --- | --- | --- |
| 1 | Yu jia he Xiang, Lushan District, Jiujiang, Jiangxi, China | 25°08′ | 117°02′ | 16,684,222 | 2,462,787,842 | MW375711 |
| 3 | Longyan, Fujian, China | 25°08′ | 117°02′ | 16,684,222 | 2,462,787,842 | MW375708 |
| 3 | Baise, Guangxi, China | 23°90′ | 106°62′ | 16,865,796 | 2,452,792,396 | MW375709 |
| 4 | Wanyuan, Dazhou, Sichuan, China | 32°08′ | 108°03′ | 19,807,966 | 2,934,684,341 | MW375710 |
| 5 | Jin zhu zhen, Sui chang County, Zhejiang, China | 28°61′ | 119°05′ | 25,887,772 | 3,861,873,758 | MW375707 |

**Supplementary Table 2.** PCR primers designed according to the hotspots within five samples of *T. hemsleyanum*

| **Hotspots** | **Primer forward (5’ to 3’)** | **Primer reverse (5’ to 3’)** |
| --- | --- | --- |
| *ndhC-trnV* | GAATTAATTCTAACTCCCACATGAT | ATTGATAAGGTGAATACCCAGTC |
| *accD* | TTACAACTCCGTCTACCCCATCC | TTCGTTTACTGGAACTCCCTCGT |
| *trnL* | GTACTTTATTTGCTGGGTTACGG | GTCTTCCTCCACTAGCAGGTTTT |
| *trnN* | CTAGTTGTAAATAGCCCCAGGAC | TAGACTAGAAACGACATCCCTTA |
| *ndhD-psaC* | GAATTAATTCTAACTCCCACATGAT | ATCATGTGGGAGTTAGAATTAATTC |

**Supplementary Table 3.** Codon usage bias in all the protein-coding genes of the chloroplast genomes of the eight Vitaceae plants

| **Amino acid** | **Codon** | **Numbers and RSCU** | | | | | |
| --- | --- | --- | --- | --- | --- | --- | --- |
|  |  | *T. hemsleyanum* | | | *T. planicaule* | *A. japonica* | *V. vinifera* |
|  |  | Jiangxi and Sichuan | Fujian and Guangxi | Zhejiang |  |  |  |
| **Ala** | GCU | 635/1.81 | 636/1.82 | 636/1.82 | 647/1.82 | 641/1.85 | 647/1.82 |
|  | GCC | 225/0.64 | 225/0.64 | 225/0.64 | 227/0.64 | 221/0.64 | 235/0.66 |
|  | GCA | 384/1.10 | 384/1.10 | 384/1.10 | 388/1.10 | 382/1.10 | 388/1.09 |
|  | GCG | 156/0.45 | 156/0.45 | 156/0.45 | 153/0.43 | 140/0.40 | 153/0.43 |
| **Arg** | AGA | 516/1.89 | 516/1.89 | 516/1.89 | 524/1.90 | 479/1.82 | 511/1.87 |
|  | AGG | 178/0.65 | 178/0.65 | 178/0.65 | 179/0.65 | 181/0.69 | 182/0.67 |
|  | CGU | 356/1.30 | 356/1.30 | 356/1.30 | 357/1.29 | 352/1.34 | 358/1.31 |
|  | CGC | 99/0.36 | 99/0.36 | 99/0.36 | 100/0.36 | 89/0.34 | 96/0.35 |
|  | CGA | 370/1.35 | 370/1.35 | 370/1.35 | 376/1.36 | 360/1.37 | 372/1.36 |
|  | CGG | 122/0.45 | 122/0.45 | 122/0.45 | 121/0.44 | 116/0.44 | 121/0.44 |
| **Asn** | AAU | 1013/1.55 | 1014/1.55 | 1014/1.55 | 1036/1.55 | 985/1.55 | 1015/1.54 |
|  | AAC | 296/0.45 | 297/0.45 | 297/0.45 | 300/0.45 | 283/0.45 | 304/0.46 |
| **Asp** | GAU | 879/1.60 | 879/1.60 | 879/1.60 | 884/1.59 | 867/1.60 | 891/1.61 |
|  | GAC | 223/0.40 | 223/0.40 | 223/0.40 | 225/0.41 | 214/0.40 | 219/0.39 |
| **Cys** | UGU | 231/1.43 | 231/1.44 | 231/1.43 | 233/1.43 | 224/1.45 | 237/1.46 |
|  | UGC | 91/0.57 | 89/0.56 | 91/0.57 | 94/0.57 | 84/0.55 | 88/0.54 |
| **Gln** | CAA | 735/1.55 | 735/1.55 | 735/1.55 | 736 1.54 | 728/1.56 | 748/1.54 |
|  | CAG | 214/0.45 | 214/0.45 | 214/0.45 | 222 0.46 | 207/0.44 | 222/0.46 |
| **Glu** | GAA | 1040/1.51 | 1040/1.51 | 1040/1.51 | 1059/1.50 | 1015/1.49 | 1060/1.50 |
|  | GAG | 342/0.49 | 342/0.49 | 342/0.49 | 349/0.50 | 346/0.51 | 352/0.50 |
| **Gly** | GGU | 608/1.35 | 608/1.34 | 608/1.35 | 614/1.35 | 597/1.36 | 617/1.35 |
|  | GGC | 167/0.37 | 169/0.37 | 167/0.37 | 169/0.37 | 153/0.35 | 160/0.35 |
|  | GGA | 740/1.64 | 740/1.64 | 740/1.64 | 746/1.63 | 733/1.66 | 762/1.67 |
|  | GGG | 292/0.65 | 293/0.65 | 292/0.65 | 297/0.65 | 279/0.63 | 288/0.63 |
| **Ile** | AUU | 1117/1.47 | 1118/1.47 | 1118/1.47 | 1130/1.48 | 1090/1.45 | 1123/1.45 |
|  | AUC | 476/0.63 | 478/0.63 | 478/0.63 | 477/0.62 | 468/0.62 | 485/0.63 |
|  | AUA | 684/0.90 | 681/0.90 | 681/0.90 | 689/0.90 | 693/0.92 | 710/0.92 |
| **Leu** | UUA | 827/1.79 | 826/1.79 | 826/1.79 | 836/1.79 | 819/1.80 | 853/1.81 |
|  | UUG | 580/1.25 | 579/1.25 | 579/1.25 | 576/1.23 | 570/1.26 | 587/1.25 |
|  | CUU | 574/1.24 | 575/1.24 | 575/1.24 | 589/1.26 | 571/1.26 | 589/1.25 |
|  | CUC | 193/0.42 | 193/0.42 | 193/0.42 | 197/0.42 | 182/0.40 | 190/0.40 |
|  | CUA | 399/0.86 | 399/0.86 | 399/0.86 | 402/0.86 | 388/0.85 | 399/0.85 |
|  | CUG | 203/0.44 | 202/0.44 | 202/0.44 | 200/0.43 | 194/0.43 | 203/0.43 |
| **Lys** | AAA | 1031/1.47 | 1031/1.47 | 1032/1.47 | 1036/1.47 | 988/1.45 | 1039/1.46 |
|  | AAG | 373/0.53 | 371/0.53 | 371/0.53 | 378/0.53 | 371/0.55 | 389/0.54 |
| **His** | CAU | 492/1.52 | 492/1.52 | 492/1.52 | 492/1.52 | 479/1.50 | 485/1.49 |
|  | CAC | 156/0.48 | 156/0.48 | 156/0.48 | 154/0.48 | 161/0.50 | 167/0.51 |
| **Met** | AUG | 634/1.00 | 636/1.00 | 636/1.00 | 645/1.00 | 618/1.00 | 647/1.00 |
| **Pro** | CCU | 443/1.59 | 442/1.59 | 442/1.59 | 449/1.61 | 436/1.61 | 451/1.60 |
|  | CCC | 200/0.72 | 200/0.72 | 200/0.72 | 197/0.70 | 191/0.71 | 198/0.70 |
|  | CCA | 320/1.15 | 321/1.15 | 321/1.15 | 316/1.13 | 306/1.13 | 327/1.16 |
|  | CCG | 153/0.55 | 152/0.55 | 152/0.55 | 156/0.56 | 150/0.55 | 152/0.54 |
| **Phe** | UUU | 938/1.25 | 941/1.25 | 941/1.25 | 958/1.25 | 932/1.26 | 954/1.27 |
|  | UUC | 560/0.75 | 560/0.75 | 560/0.75 | 569/0.75 | 542/0.74 | 549/0.73 |
| **Ser** | UCU | 591/1.70 | 589/1.69 | 590/1.70 | 588/1.67 | 579/1.69 | 593/1.71 |
|  | UCC | 333/0.96 | 333/0.96 | 333/0.96 | 342/0.97 | 334/0.98 | 329/0.95 |
|  | UCA | 444/1.28 | 444/1.28 | 444/1.28 | 450/1.28 | 437/1.28 | 441/1.27 |
|  | UCG | 188/0.54 | 187/0.54 | 187/0.54 | 194/0.55 | 183/0.54 | 193/0.56 |
|  | AGU | 406/1.17 | 406/1.17 | 406/1.17 | 415/1.18 | 402/1.18 | 406/1.17 |
|  | AGC | 126/0.36 | 126/0.36 | 126/0.36 | 126/0.36 | 117/0.34 | 118/0.34 |
| **Ter** | UAA | 42/1.43 | 42/1.43 | 42/1.43 | 43/1.50 | 41/1.46 | 40/1.36 |
|  | UAG | 23/0.78 | 23/0.78 | 23/0.78 | 23/0.80 | 22/0.79 | 23/0.78 |
|  | UGA | 23/0.78 | 23/0.78 | 23/0.78 | 20/0.70 | 21/0.75 | 25/0.85 |
| **Thr** | ACU | 542/1.61 | 534/1.61 | 542/1.61 | 547/1.60 | 529/1.59 | 554/1.61 |
|  | ACC | 240/0.71 | 239/0.71 | 239/0.71 | 244/0.71 | 242/0.73 | 250/0.73 |
|  | ACA | 426/1.26 | 426/1.26 | 426/1.27 | 435/1.27 | 415/1.25 | 422/1.23 |
|  | ACG | 140/0.42 | 140/0.42 | 140/0.42 | 145/0.42 | 145/0.44 | 150/0.44 |
| **Trp** | UGG | 471/1.00 | 469/1.00 | 471/1.00 | 472/1.00 | 461/1.00 | 470/1.00 |
| **Tyr** | UAU | 794/1.62 | 795/1.62 | 795/1.62 | 813/1.63 | 783/1.62 | 815/1.63 |
|  | UAC | 188/0.38 | 188/0. 38 | 188/0. 38 | 187/0.37 | 184/0.38 | 188/0.37 |
| **Val** | GUU | 501/1.40 | 500/1.40 | 500/1.40 | 507/1.39 | 493/1.40 | 499/1.38 |
|  | GUC | 183/0.51 | 185/0.52 | 184/0.51 | 190/0.52 | 180/0.51 | 192/0.53 |
|  | GUA | 533/1.49 | 532/1.49 | 533/1.49 | 541/1.48 | 525/1.50 | 551/1.52 |
|  | GUG | 215/0.60 | 212/0.59 | 213/0.60 | 221/0.61 | 206/0.59 | 208/0.57 |

**Supplementary Table 4.** Divergency in protein coding sequences of *T. hemsleyanum* in five regions.

| **Gene** | **Divergency of Codons and Amino acid** | | |
| --- | --- | --- | --- |
|  | Jiangxi and Sichuan | Fujian and Guangxi | Zhejiang |
| *psbA* | GTG(V) | TTG(L) | TTG(L) |
| *matK* | CCG(L) | CCA(P) | CCA(P) |
| *rpoC2* | CTG(L) | CTT(L) | CTT(L) |
| *psaA* | TCT(S) | GCT(A) | GCT(A) |
| *rps4* | TCG(S) | TCG(S) | TCT(S) |
| *atpB* | ACT(T) | ACT(T) | AAA(K) |
| *rbcL* | GTT(V) | ATT(I) | ATT(I) |
| *accD* | AAG(K) | ATG(M) | ATG(M) |
| *accD* | AAA(K) | CAA(Q) | CAA(Q) |
| *accD* | AAG(K) | AAC(N) | AAC(N) |
| *ycf4* | CAA(Q) | AAA(K) | AAA(K) |
| *petA* | GTA(V) | GTC(V) | GTC(V) |
| *petD* | ACC(T) | AAC(N) | AAC(N) |
| *rps11* | AAC(N) | AAT(N) | AAT(N) |
| *rpl22* | GTG(V) | ATG(M) | ATG(M) |
| *ycf2* | TTG(L) | TTT(F) | TTT(F) |
| *ycf2* | TGC(C) | GGC(G) | TGC(C) |
| *ndhB* | ATA(I) | ATC(I) | ATC(I) |
| *ccsA* | TTA(L) | TTT(F) | TTT(F) |
| *ccsA* | CCT(P) | ACT(T) | ACT(T) |
| *ndhD* | GTG(V) | GGG(G) | GTG(V) |
| *rps15* | ATA(I) | GTA(V) | GTA(V) |
| *ycf1* | GTA(V) | GTC(V) | GTA(V) |
| *ycf1* | TCT(S) | TAT(Y) | TAT(Y) |
| *ndhB* | ATA(I) | ATC(I) | ATC(I) |
| *ycf2* | TTG(L) | TTT(F) | TTT(F) |
| *ycf2* | TGC(C) | GGC(G) | TGC(C) |

| Amino acid conversion | Edited position | Number and percentage | | | |
| --- | --- | --- | --- | --- | --- |
|  |  | *T. hemsleyanum* (Jiangxi) | *T. planicaule* | *A. japonica* | *V. vinifera* |
| S-L | Second nucleotide | 30/42.3% | 30/42.3% | 30/41.7% | 30/42.9% |
| P-L | Second nucleotide | 6/8.5% | 8/11.3% | 8/11.1% | 8/11.2% |
| H-Y | First nucleotide | 7/9.9% | 6/8.5% | 6/8.3% | 6/8.6% |
| L-F | First nucleotide | 6/8.5% | 6/8.5% | 7/9.7% | 6/8.6% |
| S-F | Second nucleotide | 6/8.5% | 5/7.0% | 6/8.3% | 5/7.1% |
| T-M | Second nucleotide | 5/7.0% | 5/7.0% | 4/5.6% | 4/5.7% |
| A-V | Second nucleotide | 4/5.6% | 4/5.6% | 5/6.9% | 4/5.7% |
| P-S | First nucleotide | 2/2.8% | 2/2.8% | 2/2.8% | 2/2.9% |
| R-W | First nucleotide | 1/1.4% | 1/1.4% | 1/1.4% | 1/1.4% |
| T-I | Second nucleotide | 3/4.2% | 3/4.2% | 2/2.8% | 3/4.3% |
| R-C | First nucleotide | 1/1.4% | 1/1.4% | 1/1.4% | 1/1.4% |

**Supplementary Table 5.** Amino acid conversion frequency of protein coding genes of *T. hemsleyanum* and three Vitaceae species

**Supplementary Table 6.** Variable bases of *trnL* and *trnN* sequences of *T. hemsleyanum* from different regions.

| Samples from different provinces | variable bases’ location | | | | | | | | | |
| --- | --- | --- | --- | --- | --- | --- | --- | --- | --- | --- |
|  | *trnL* | | | | | | *trnN* | | | |
|  | 165bp | 166bp | 167bp | 168bp | 671bp | 1036bp | 164bp | 165bp | 166bp | 167bp |
| Jiangxi | A | T | C | A | T | G | A | A | A | G |
| Zhejiang  (01-03; 07-10) | A | T | C | A | T | G | A | A | A | G |
| Zhejiang (04-06) | T | G | A | T | T | G | A | A | A | G |
| Zhejiang (11-15) | T | G | A | T | C | G | A | A | A | G |
| Fujian | A | T | C | A | T | G | A | A | A | G |
| Guangxi | A | T | C | A | T | G | A | A | A | G |
| Sichuan | A | T | C | A | T | T | C | T | T | T |
| Guangdong | A | T | C | A | T | G | A | A | A | G |
| Genbank | A | T | C | A | T | G | A | A | A | G |
